# Supplementary material for: Superior ferroelectricity and nonlinear optical response in a hybrid germanium iodide hexagonal perovskite
Source: Nat Commun. 2023 May 19;14:2863. doi: 10.1038/s41467-023-38590-7 (PMC10199078; doi:10.1038/s41467-023-38590-7)
Supplement: Supplementary file 1 — Supplementary Information [file 41467_2023_38590_MOESM1_ESM.docx]

**Supporting Information**

**Superior ferroelectricity and nonlinear optical response in a hybrid germanium iodide hexagonal perovskite**

Kun Ding^1,2,#^, Haoshen Ye^3,#^, Changyuan Su^1,#^, Yu-An Xiong^1^, Guowei Du^1^, Yu-Meng You^1^, Zhi-Xu Zhang^1,2^, Shuai Dong^3^,Yi Zhang^2^ and Da-Wei Fu^2^

^1^ Jiangsu Key Laboratory for Science and Applications of Molecular Ferroelectrics, Southeast University, Nanjing 211189, China

^2^ Institute for Science and Applications of Molecular Ferroelectrics, Key Laboratory of the Ministry of Education for Advanced Catalysis Materials, Zhejiang Normal University, Jinhua, China

^3^ Key Laboratory of Quantum Materials and Devices of Ministry of Education, School of Physics, Southeast University, Nanjing 21189, China

^#^ These authors contributed equally: Kun Ding, Haoshen Ye, Changyuan Su

Correspondence and requests for materials should be addressed to Z.-X. Z. (email: [zhixu@seu.edu.cn](mailto:zhixu@seu.edu.cn)); S.D. (email: [sdong@seu.edu.cn](mailto:sdong@seu.edu.cn)); Y.Z. (email: [yizhang1980@seu.edu.cn](mailto:yizhang1980@seu.edu.cn)) or to D.-W. F. (email: [dawei@seu.edu.cn](mailto:dawei@seu.edu.cn))

**This PDF file includes:**

Supplementary Notes 1 - 4

Supplementary Figures 1 - 20

Supplementary Tables 1 - 9

Supplementary References 1 - 58

**Contents of Supporting Information**

| **Supplementary Fig. 1** | The Strategy for designing Ge-based molecular ferroelectric materials. | S4 |
| --- | --- | --- |
| **Supplementary Note 1** | More details of synthesis and crystal growth | S4 |
| **Supplementary** **Fig. 2** | Powder X-ray diffraction patterns of DMAGeI_3_ in different test conditions. | S5 |
| **Supplementary Fig. 3** | TGA curve of DMAGeI_3_. | S6 |
| **Supplementary Fig. 4** | Differential Scanning Calorimeter (DSC) curves of DMAGeI_3_. | S6 |
| **Supplementary Fig. 5** | The crystal structural analysis of DMAGeI_3_. | S7 |
| **Supplementary Fig. 6** | Comparison of DMA cations. | S7 |
| **Supplementary Fig. 7** | Comparison of bond lengths and bond angles of GeI_6_ octahedron. | S8 |
| **Supplementary Table 1** | Crystal data and structure refinement details for compound DMAGeI_3_. | S8 |
| **Supplementary Table 2** | Selected bond lengths (Å) and angles (°) for compounds DMAGeI_3_ at 293 and 373 K, respectively. | S9 |
| **Supplementary Table 3** | The Ge-I bond length (Å) and I-Ge-I bond angle (^o^) of the GeI_6_ octahedron at 293 and 373 K, respectively. | S9 |
| **Supplementary Note 2** | More details of ferroelectric characterization. | S9 |
| **Supplementary Fig. 8** | The particle size (diameter) dependence of SHG intensity for both DMAGeI_3_ and KDP under 1064 nm laser radiation. | S11 |
| **Supplementary Fig. 9** | Schematic of the experimental setup to illustrate the sample orientation with respect to the stage geometry and light propagation direction. | S12 |
| **Supplementary Fig. 10** | a, Experimental diagram and photograph of crystal. b, Schematic of the non-linear optical converter. | S12 |
| **Supplementary Table 4** | Comparison of SHG signal strength between reported well-known inorganic nonlinear optical material and organic-inorganic hybrid perovskite and DMAGeI_3_ in this work. | S13 |
| **Supplementary Fig. 11** | Temperature-dependent dielectric real part (*ε′*) of DMAGeI_3_ measured at various frequencies. | S14 |
| **Supplementary Fig. 12** | Crystal process of DMAGeI_3_, cut perpendicular to the *c*-axis. | S14 |
| **Supplementary Fig. 13** | Photos of *d*_33_ meter. | S14 |
| **Supplementary Table 5** | Comparison of the maximum dielectric real part (*ε′*) at *T*_c_ of organic-inorganic hybrid perovskite ferroelectrics and DMAGeI_3_ in this work. | S15 |
| **Supplementary Table 6** | Comparison of spontaneous polarization *P*_s_ in a variety of materials and DMAGeI_3_ in this work. | S16 |
| **Supplementary Table 7** | Comparison of coercive field *E*_c_ in a variety of materials and DMAGeI_3_ in this work. | S17 |
| **Supplementary Fig. 14** | Domain structures in the (010) plane of DMAGeI_3_. | S17 |
| **Supplementary Fig. 15** | The measurements of polarization reversal for DMAGeI_3_ in lateral PFM. | S18 |
| **Supplementary Fig. 16** | Structural illustration of the 3D perovskite DMAGeCl_3_ and DMAGeBr_3_ viewed along the *b*-axis. | S18 |
| **Supplementary Table 8** | Crystal data and structure refinement details compound DMAGeCl_3_ and DMAGeBr_3_. | S19 |
| **Supplementary Table 9** | Selected bond lengths (Å) and angles (°) for compounds DMAGeCl_3_ and DMAGeBr_3_ at 293 K, respectively. | S19 |
| **Supplementary Note 3** | More details of DFT calculations. | S19 |
| **Supplementary Fig. 17** | The test of functionals and van der Waals corrections | S20 |
| **Supplementary Fig. 18** | DFT electronics properties of DMAGeI_3_. | S21 |
| **Supplementary Fig. 19** | Electron density difference between DMAGeI_3_ and extracted parts with holes/electrons added | S21 |
| **Supplementary Note 4** | More details of SHG calculations. | S21 |
| **Supplementary Fig. 20** | SHG analysis. | S23 |

**
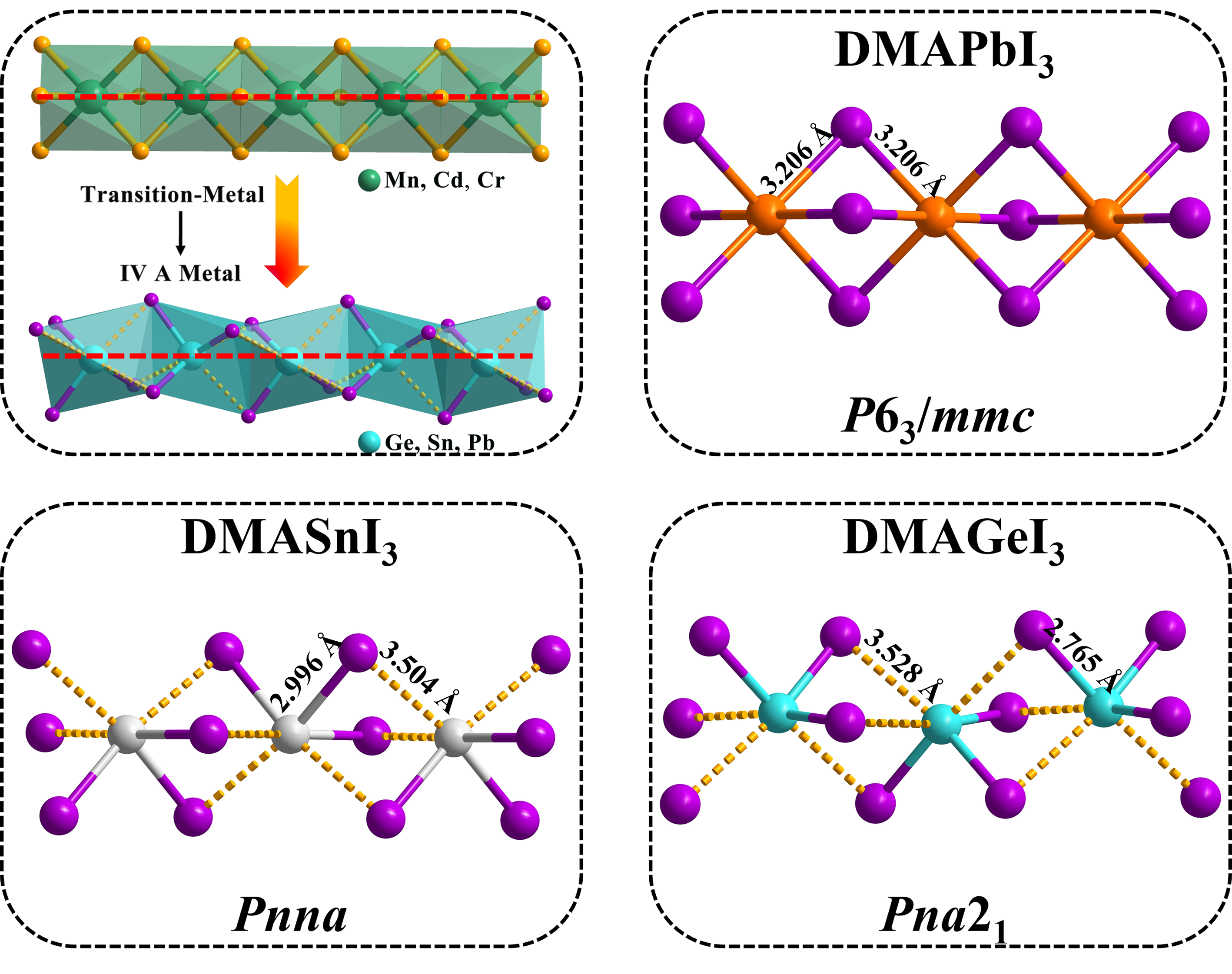
**

**Supplementary Figure 1 | The Strategy for designing Ge-based molecular ferroelectric materials.** The structures of DMAPbI_3_ ^[1]^, DMASnI_3_ ^[2]^, and DMAGeI_3_ are obtained at 293 K.

**Supplementary Note 1 | More details of synthesis and crystal growth.**

*N, N*-Dimethylformamide (DMF, 97%, Macklin), GeO_2_ (99%, Macklin), HBr (40%, Macklin), HCl (36%, Adamas), H_3_PO_2_ (50 wt.% in H_2_O, Macklin) and HI (50.0%, Macklin). All chemicals were commercially available and were used without further purification.

DMAGeCl_3_ was prepared by dissolving 2 mmol of GeO_2_ in a mixed solution of 10 mL HCl, 5 mL H_3_PO_2_ and 5 mL DMF. First, 2 mmol (0.209 g) GeO_2_ was slowly added into the mixed solution of 10 mL HCl and 5 mL H_3_PO_2_. A clear solution was obtained after strong stirring for 2 h at 373 K. Subsequently, 5 mL DMF was added to the solution which continued to stir for 1 h. After the reaction mixture was cooled to room temperature and filtered, the colorless solution was kept at room temperature for slow evaporation. The colorless block crystals were obtained about a few days later. Following a similar synthetic procedure, the colorless block crystals DMAGeBr_3_ could also be obtained (Supplementary Fig. 16). The isostructural compounds DMAGeCl_3_ and DMAGeBr_3_ crystallize in the orthorhombic system of space group *Pbca* at room temperature. More detailed structural information is summarized in Supplementary Tables 8-9.

However, the DMAGeI_3_ crystals could not be obtained under the above experimental conditions. We successfully obtained DMAGeI_3_ single crystals through the hydrothermal method. The GeO_2_ (2 mmol) was dissolved in a mixed solution of 3 mL HI, 3 mL H_3_PO_2_, and 3 mL DMF. The prepared suspension was then transferred into 25 mL Teflon-lined stainless-steel autoclaves and maintained at 363 K for 6 h. Then, the saturated solution was slowly cooled with a temperature rate of 5 K/day. Finally, the yellow rod-shaped crystals were successfully obtained after cooling the autoclave to room temperature. The DMAGeI_3_ crystallizes in a polar space group *Pna*2_1_ at room temperature. More detailed structural information is summarized in Supplementary Tables 1-2.


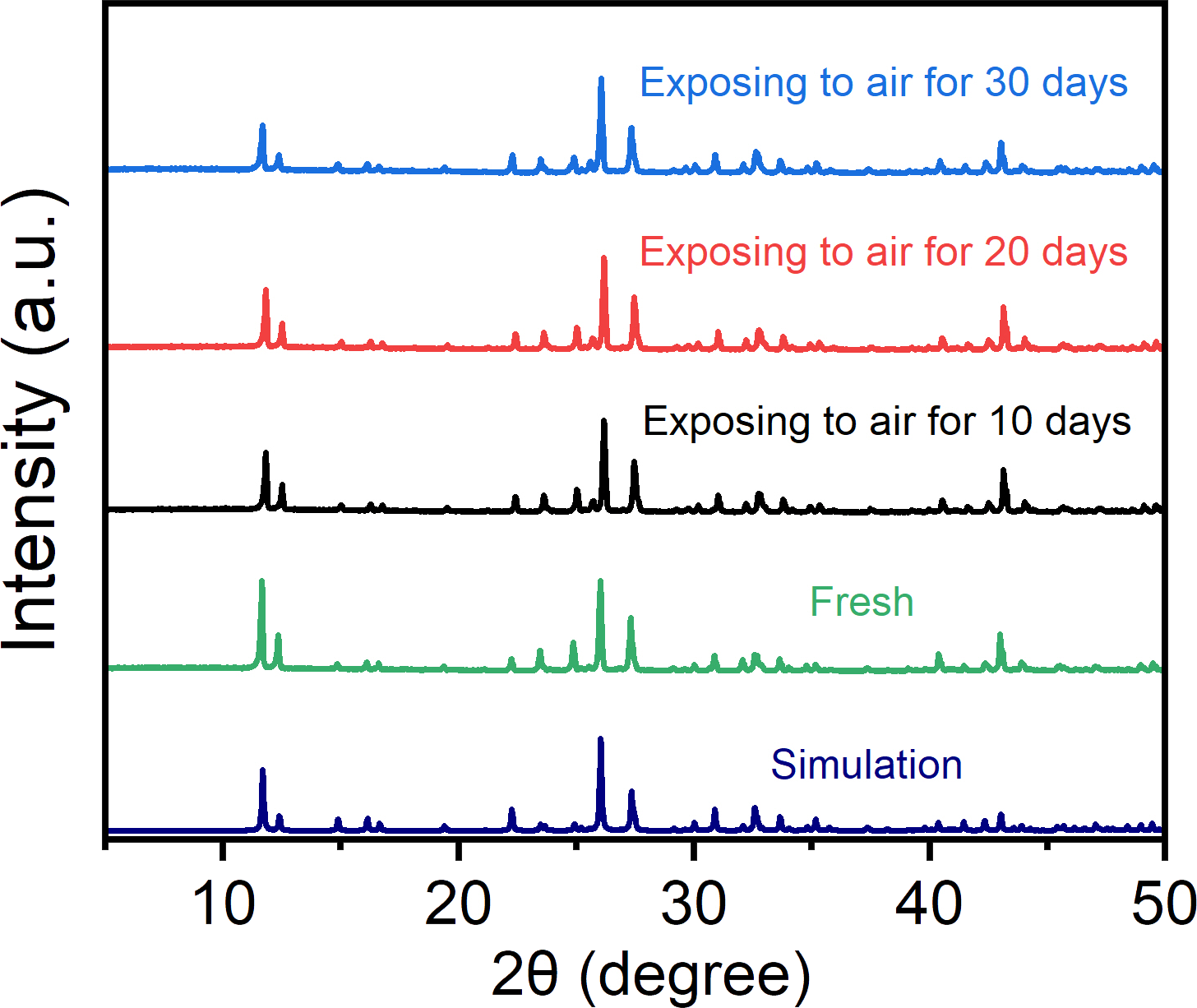


**Supplementary Fig. 2 | Powder X-ray diffraction patterns of DMAGeI_3_ in different test conditions.**


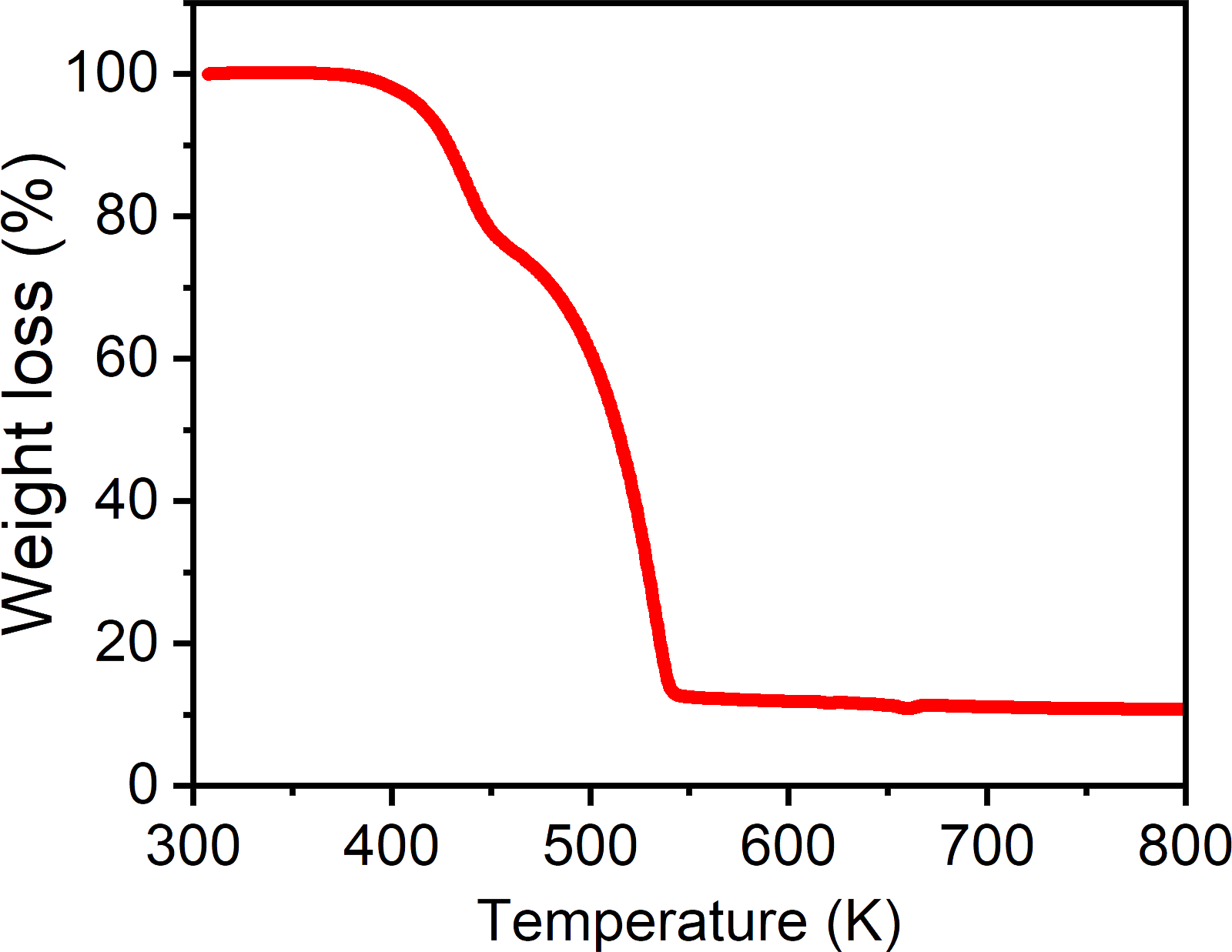


**Supplementary Fig. 3 | TGA curve of DMAGeI_3_.**


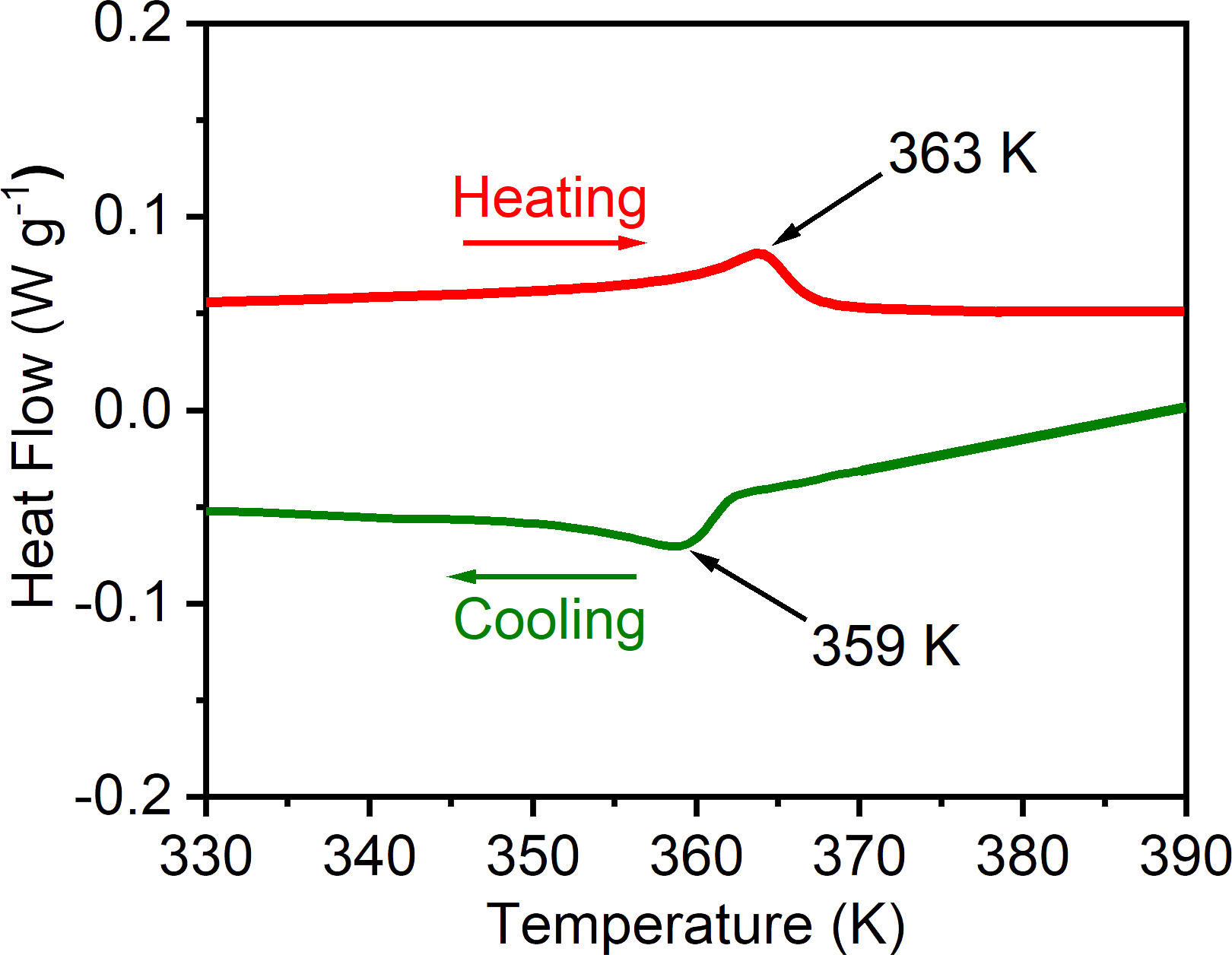


**Supplementary** **Fig. 4 | Differential Scanning Calorimeter (DSC) curves of DMAGeI_3_.**

**
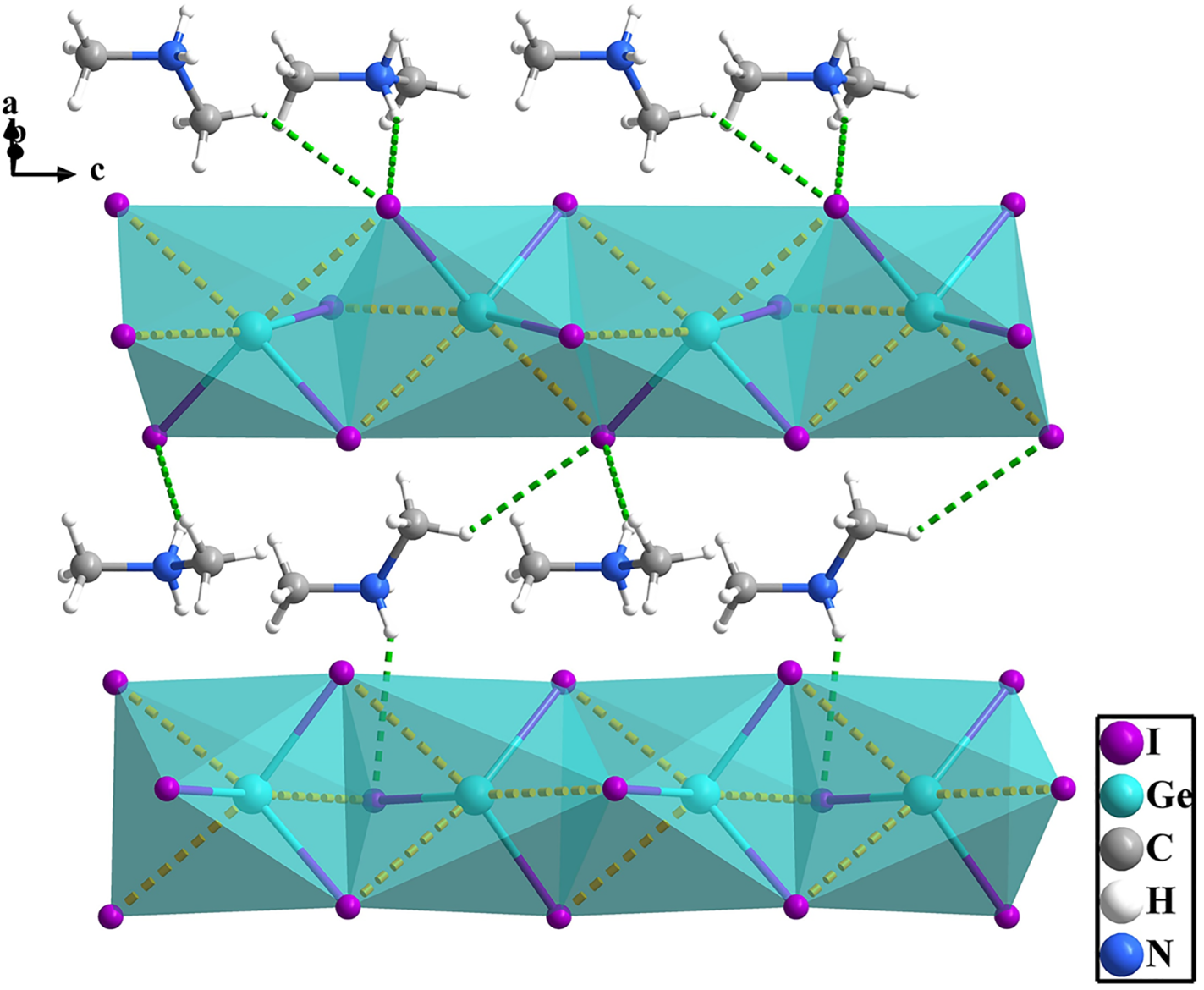
**

**Supplementary Fig. 5 | The crystal structural analysis of DMAGeI_3_.** The N-H···I hydrogen-bonding interactions between organic cations and inorganic perovskite frameworks of DMAGeI_3_ at 293 K.

**
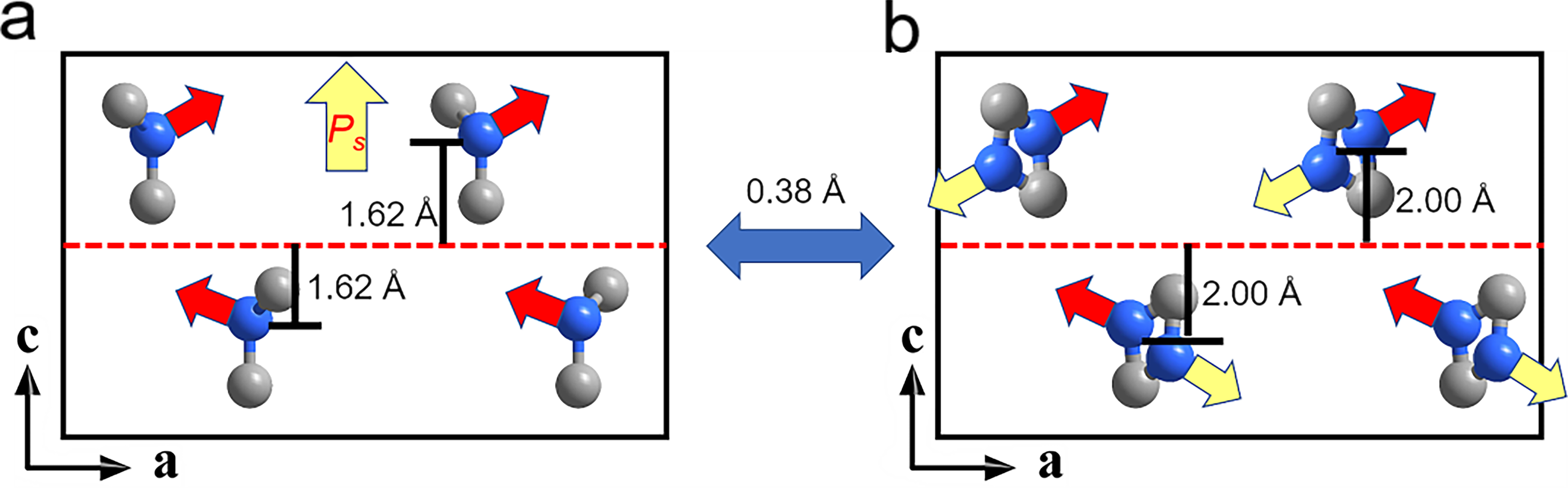
**

**Supplementary Fig. 6 | Comparison of DMA cations. a-b**, The relative displacement of DMA cations along the *c*-axis at 293 K, 373 K. The red dashed lines indicate the horizontal center position in the unit cell and the local dipoles are indicated by arrows.

**
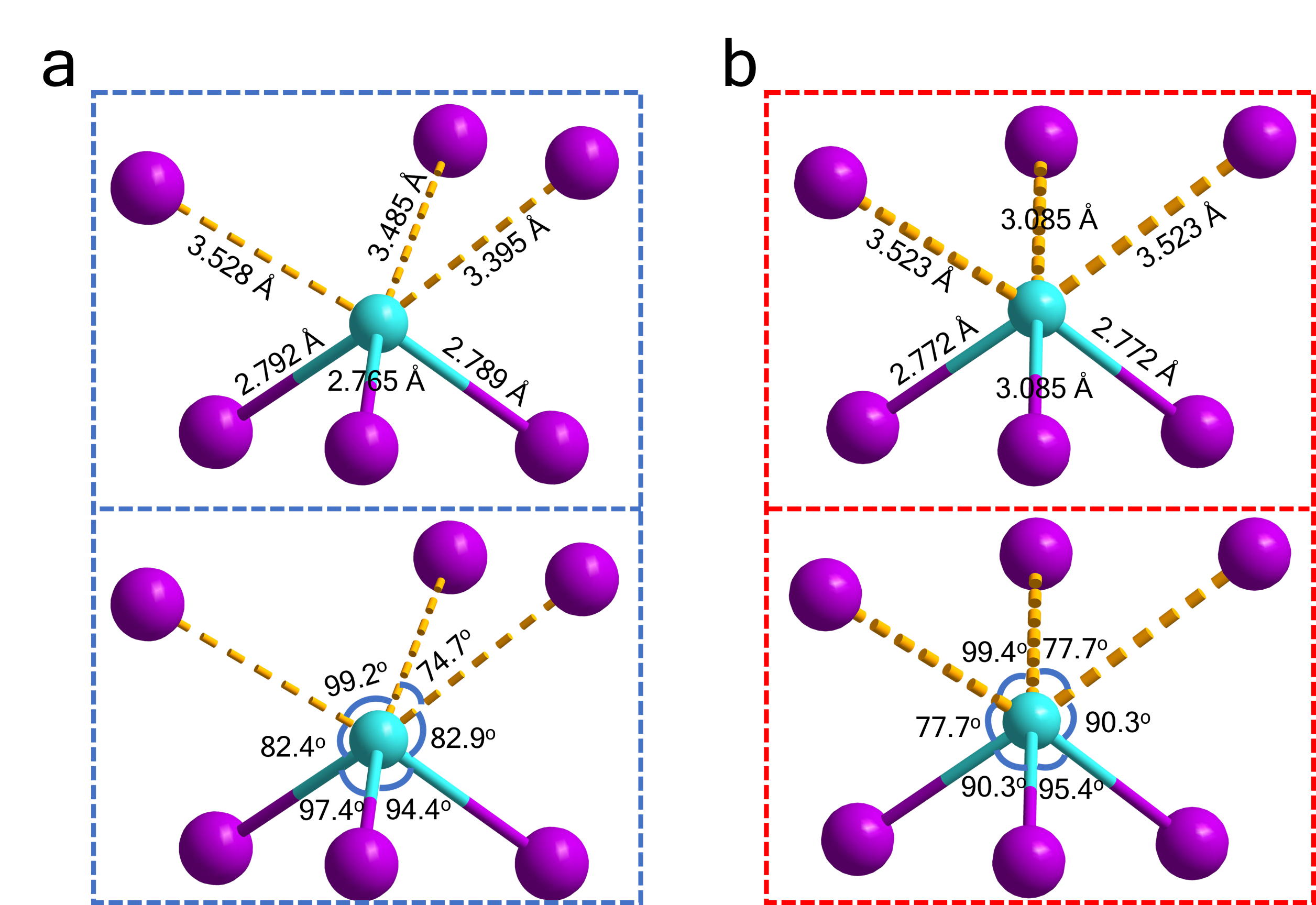
**

**Supplementary Fig. 7 | Comparison of bond lengths and bond angles of GeI_6_ octahedron. a-b**, 293 K and 373 K, respectively.

**Supplementary Table 1 | Crystal data and structure refinement details for compound DMAGeI_3_.**

| DMAGeI_3_ | 223 K 253 K 293 K 373 K | | | | |
| --- | --- | --- | --- | --- | --- |
| Empirical formula | | C_2_H_8_NGeI_3_ | C_2_H_8_NGeI_3_ | C_2_H_8_NGeI_3_ | C_2_H_8_NGeI_3_ |
| Formula weight | | 499.40 | 499.40 | 499.40 | 499.40 |
| Crystal system | | orthorhombic | orthorhombic | orthorhombic | orthorhombic |
| Space group | | *Pna*2_1_ | *Pna*2_1_ | *Pna*2_1_ | *Pnan* |
| a/Å | | 14.1481(4) | 14.1793(7) | 14.2829(10) | 14.5493(6) |
| b/Å | | 8.8837(3) | 8.9119(4) | 8.9305(8) | 8.9096(4) |
| c/Å | | 7.9434(3) | 7.9572(4) | 7.9828(6) | 8.0080(3) |
| *α*/° | | 90 | 90 | 90 | 90 |
| *β*/° | | 90 | 90 | 90 | 90 |
| *γ*/° | | 90 | 90 | 90 | 90 |
| Volume/Å^3^ | | 998.39(6) | 1005.51(8) | 1018.23(14) | 1038.07(7) |
| Z | | 4 | 4 | 4 | 4 |
| *F* (000) | | 872.0 | 872.0 | 868.0 | 840.0 |
| Radiation | | Mo Kα | Mo Kα | Mo Kα | Mo Kα |
| absorption correction | | emiempirical | emiempirical | emiempirical | emiempirical |
| GOF | | 1.070 | 1.100 | 1.047 | 1.101 |
| *R*_1_ | | 0.0774 | 0.0789 | 0.0822 | 0.0440 |
| *wR*_2_ | | 0.2024 | 0.2147 | 0.1939 | 0.1143 |

**Supplementary Table 2 | Selected bond lengths (Å) and angles (°) for compounds DMAGeI_3_ at 293 and 373 K, respectively.**

| 293 K 373 K | | | | |
| --- | --- | --- | --- | --- |
| I001—Ge04 | 2.792 (3) |  | I001—Ge03 | 2.7722 (10) |
| I002—Ge04 | 2.765 (3) |  | I002—Ge03 | 3.0852 (7) |
| I003—Ge04 | 2.789 (3) |  | C2—N1^i^ | 1.35 (2) |
| C005—N1 | 1.52 (4) |  | C2—N1 | 1.49 (3) |
| C006—N1 | 1.45 (4) |  |  |  |
|  |  |  | I001^ii^—Ge03—I001 | 95.46 (4) |
| I002—Ge04—I001 | 97.42 (10) |  | I001^ii^—Ge03—I002 | 94.77 (2) |
| I002—Ge04—I003 | 94.51 (10) |  | N1^i^—C2—N1 | 61.7 (15) |
| I003—Ge04—I001 | 94.48 (9) |  | C2^i^—N1—C2 | 114.7 (15) |
| C006—N1—C005 | 114 (3) |  | I001—Ge03—I002 | 90.39 (2) |
|  |  |  | N1^i^—C2—N1—C2^i^ | −22 (2) |

**Supplementary Table 3 | The Ge-I bond length (Å) and I-Ge-I bond angle (^o^) of the GeI_6_ octahedron at 293 and 373 K, respectively.** The octahedron distortion parameter Δ = $\frac{1}{6}\sum_{i=1}^{6} \left( \frac{d_{i}-d}{d} \right)^{2}$, *di*, *d* are the individual bond length and the average length of the Ge-I in the [GeI_6_] octahedron. The octahedral angle variance = $\frac{1}{11}\sum_{i=1}^{12} \left( \alpha_{i}-90 \right)^{2},$ *α_i_* is the angle of I-Ge-I in the octahedron ^[3]^.

| 293 K | | | 373 K | | |
| --- | --- | --- | --- | --- | --- |
| Ge-I bond length (Å) | I-Ge-I bond angle (^o^) | | Ge-I bond length (Å) | I-Ge-I bond angle (^o^) | |
| 2.765 | 74.696 | 84.724 | 2.772 | 97.215 | 83.013 |
| 2.792 | 99.189 | 92.789 | 2.772 | 99.463 | 77.76 |
| 2.789 | 82.914 | 94.475 | 3.085 | 94.770 | 95.471 |
| 3.396 | 94.516 | 82.191 | 3.085 | 90.388 | 83.013 |
| 3.485 | 95.104 | 94.416 | 3.523 | 94.770 | 90.388 |
| 3.528 | 82.432 | 100.884 | 3.523 | 77.76 | 97.215 |

**Supplementary Note 2 | More details of ferroelectric characterization.**

Variable-temperature single-crystal X-ray diffraction data were collected on a Rigaku VarimaxTM DW diffractometer with Mo Kα radiation (λ = 0.71073 Å). Data processing with empirical absorption correction was conducted by using the CrysAlisPro 1.171.40.14e (Rigaku OD, 2018). The crystal structures were confirmed by direct methods and refined by full-matrix least-squares methods based on *F*^2^ through the OLEX2 and SHELXTL (version 2018) software package. All non-hydrogen atoms were refined anisotropically and all hydrogen atoms were generated geometrically in suitable positions.

The second harmonic generation (SHG) experiments were performed on an Edinburgh FLS 920 by using an unexpanded laser beam with low divergence (pulsed Nd: YAG at a wavelength of 1064 nm, 5 ns pulse duration, 1.6 MW peak power, 10 Hz repetition rate) in the temperature range of 300-380 K. Furthermore, the SHG phase matching tests were performed with a particle size range of 62-375 μm at room temperature (Supplementary Fig. 8).

To derive the non-linear susceptibility, the SHG intensity anisotropy was investigated by using Witec alpha 300 on single-crystal surfaces. SHG measurements were performed in the Imaging Facilities of Molecular Foundry at LBNL. The light coming from a Coherent Chameleon Ultra II Ti: sapphire laser (700 to 1064 nm) with a pulse width of 150 fs and a repetition rate of 50 MHz was applied for the SHG spectroscopic and microscopic measurement. The incident light (frequency ω) was coupled into the microscope using a dichroic mirror (reflective for the light of frequency and transparent for frequency 2ω), then was focused onto the sample with a 100× (0.95 NA) Nikon LU Plan Apo objective and scanned across the sample for measuring SHG in reflection geometry (Supplementary Fig. 9). In the SHG polarimetry measurement, all linear polarization state of input light is available by rotating a half-wave plate (HWP) in the laser beam path. Before the light passes through HWP, there is also a polarizer to enhance the degree of linear polarization. The HWP was mounted on a motorized rotation stage and controlled by the software, allowing for a continuous and precise rotation of incident polarization *E* (φ) in the *x*-*y* plane of the laboratory coordinate system (*x*, *y*, *z*), where φ is the azimuthal angle of the fundamental light polarization.

For the dielectric measurements, the sample of DMAGeI_3_ was made with single-crystals cut perpendicular to the *a-*, *b-*, and *c*-axis respectively. Silver conduction paste deposited on the plate surfaces was used as the electrodes. The temperature-dependent dielectric constants were performed on the Tonghui TH2828A instrument under the frequency range from 1 kHz to 1 MHz with an applied electric ﬁeld of 1 V (Supplementary Fig. 11).

Single crystal electrodes of DMAGeI_3_ were prepared for the *P-E* hysteresis loop measurements by Sawyer-Tower method. To reduce the coercive force field, single crystals obtained were processed to make crystals along the polar axis (*c*-axis). Then a pair of electrodes were formed orthogonally with each other on a single crystal of 1 with silver conductive adhesive, and copper wires were used to connect with IC sockets (Supplementary Fig. 12). Ferroelectric polarization imaging and local switching studies were carried out using a resonant-enhanced PFM (MFP-3D, Asylum Research). To visualize the domain switching process, a local polarization manipulation study on the bulk crystal surface was carried out by applying a DC tip bias of ±50 V for 0.5 s. The polarization in some domains can be switched forth and back in a reproducible manner. The macroscopic piezoelectric coefficient (*d*_33_) was measured by a commercial piezometer (Piezotest, model: PM200) using the quasi-static method (Supplementary Fig. 13).


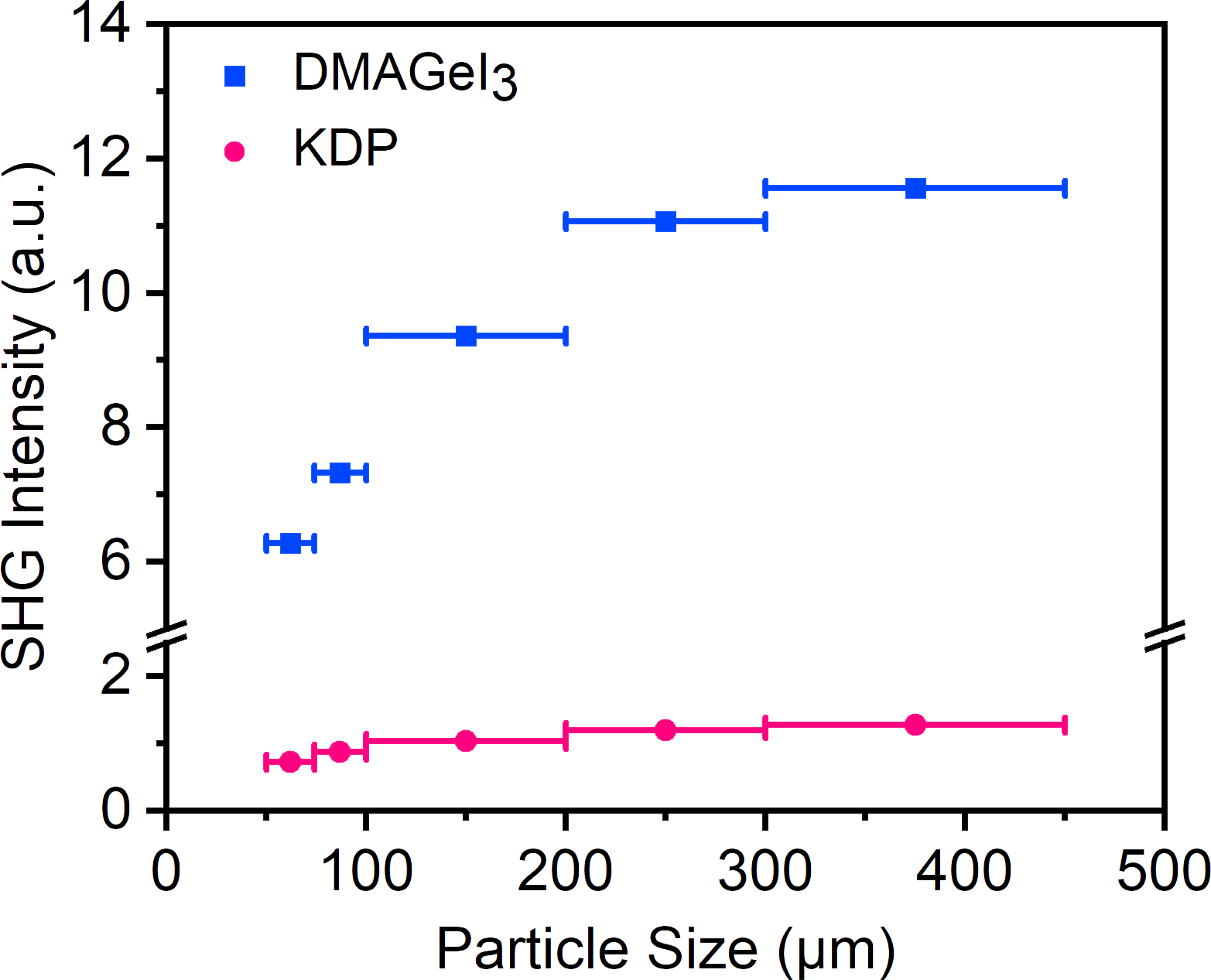


**Supplementary Fig. 8 | The particle size (diameter) dependence of SHG intensity for both DMAGeI_3_ and KDP under 1064 nm laser radiation.** The SHG intensities increase with increasing particle sizes until it attains the constant, indicating the phase matchable nature of the DMAGeI_3_.

**
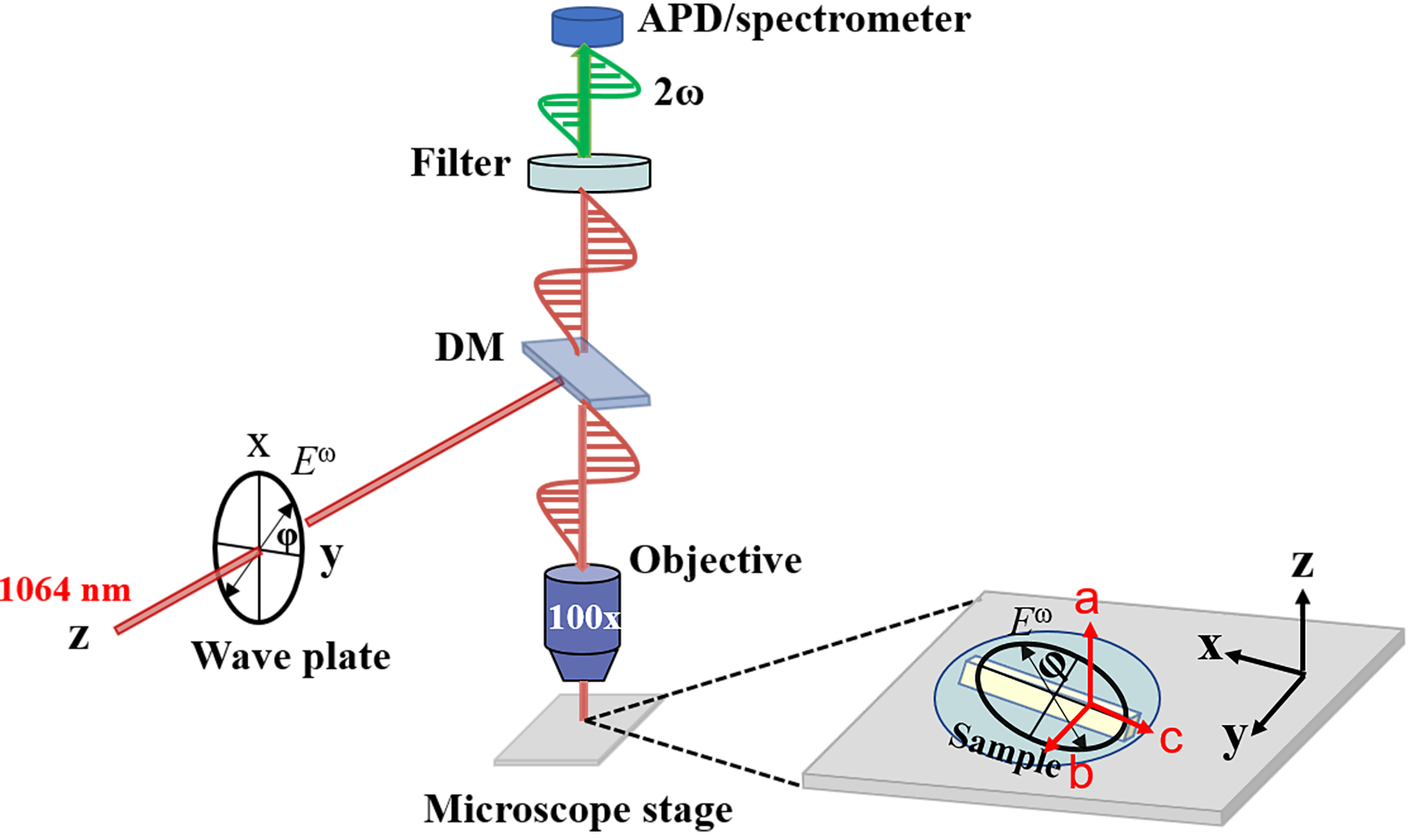
**

**Supplementary Fig. 9 | Schematic of the experimental setup to illustrate the sample orientation with respect to the stage geometry and light propagation direction.**

**
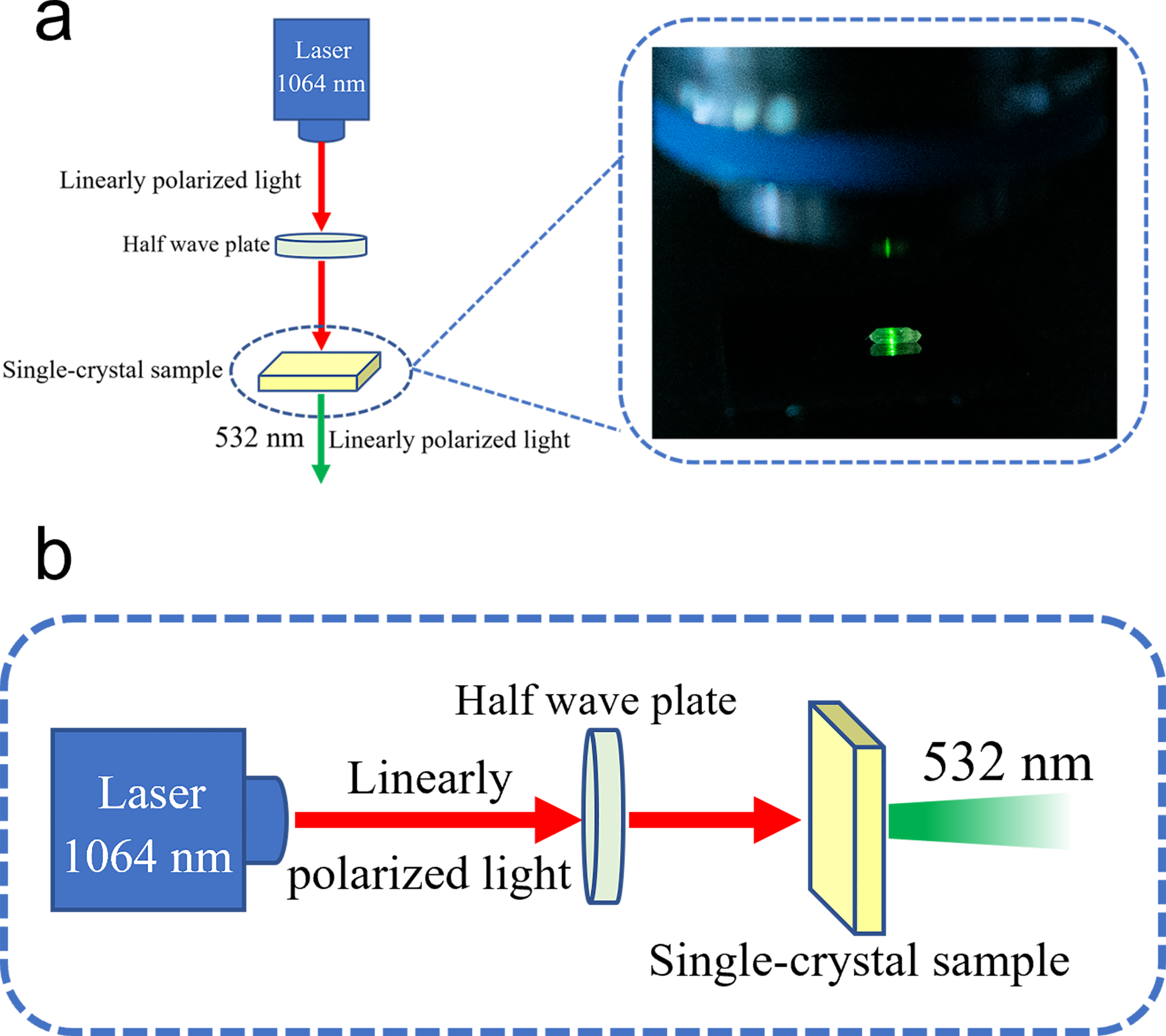
**

**Supplementary Fig. 10 | a, Experimental diagram and photograph of crystal. b, Schematic of the non-linear optical converter.** The bright green light can be clearly observed on the crystal of DMAGeI_3_ under 1064 nm linearly polarized light, which indicates that the sample generates strong second-harmonic light with wavelength of 532 nm.

**Supplementary Table 4 | Comparison of SHG signal strength between reported well-known inorganic nonlinear optical material and organic-inorganic hybrid perovskite and DMAGeI_3_ in this work.** BBO: β-BaB_2_O; LBO: LiB_3_O_5_; KABO: K_2_Al_2_B_2_O; LN: LiNbO_3_; DM: dimethylammonium; PM: phenylmethanaminium; 3-dcbm: 3,3-difluorocyclobutylammonium; 2-fbm: 2-fluorobenzylammonium; R-EQ: (R)-N-ethyl-3-quinuclidinol; DFCBA: 3,3-Difluorocyclobutylammonium; 3-HQCM: R-N-methyl-3-hydroxylquinuclidinium; *S*-1-CEM : *S*-1-(4-chlorophenyl)ethylammonium; NPD: *N*-methylpyrrolidinium; R3HQ: *R*-3-hydroxylquinuclidinium; EATMP: (2-aminoethyl)trimethylphosphanium]; TMIM: (CH_3_)_3_NCH_2_I; SPAD: 1-((2-hydroxybenzylidene)amino)pyridin-1-ium); MHy: CH_3_NH_2_NH_2_; MA: CH_3_NH_3_; FA: CH(NH_2_)_2_; TMAEA: 2-trimethylammonioethylammonium

|  | Compound | SHG intensity times vs KH_2_PO_4_ (KDP) | References |
| --- | --- | --- | --- |
| well-known  inorganic nonlinear optical material | BBO | ~4.4* | 4-7 |
|  | LBO | ~2.4* | 5,7-8 |
|  | KABO | ~1.34* | 5 |
|  | LN | ~12* | 5 |
| Organic-inorganic hybrid perovskite | [3-dcbm]_2_CuCl_4_ | ~0.25 | 9 |
|  | [2-fbm]_2_PbCl_4_ | ~0.9 | 10 |
|  | [R-EQ]PbI_3_ | 0.21 | 11 |
|  | [DFCBA]_2_CrCl_4_ | 0.25 | 12 |
|  | (3-HQCM)_2_RbCe(NO_3_)_6_ | ~0.25 | 13 |
|  | (*S*-1-CEM) _2_PbI_4_ | ~0.5 | 14 |
|  | (NPD)_3_Sb_2_Br_9_ | ~0.6 | 15 |
|  | (R_3_HQ)_4_KCe(NO_3_)_8_ | ~0.55 | 16 |
|  | (C_4_H_9_NH_3_)_2_(NH_3_CH_3_)_2_Sn_3_Br_10_ | ~1 | 17 |
|  | [(CH_3_)_2_CHCH_2_NH_3_]_2_PbCl_4_ | ~1 | 18 |
|  | (EATMP)Pb_2_Br_6_ | ~0.17 | 19 |
|  | (TMIM)PbI_3_ | ~0.65 | 20 |
|  | [CH_3_(CH_2_)_3_NH_3_]_2_(CH_3_NH_3_)Pb_2_Br_7_ | ~0.4 | 21 |
|  | (C_6_H_5_CH_2_NH_3_)_2_CsAgBiBr_7_ | ~1 | 22 |
|  | [SAPD]PbI_3_ | ~2 | 23 |
|  | MHyPbCl_3_ | ~0.03 | 24 |
|  | MHyPbBr_3_ | ~0.18 | 25 |
|  | NH(CH_3_)_3_SnCl_3_ | ~1 | 26 |
|  | NH(CH_3_)_3_SnBr_3_ | ~2.5 | 26 |
|  | MAGeBr_3_ | ~5.3 | 28 |
|  | FAGeBr_3_ | ~0.9 | 28 |
|  | FA_0.5_MA_0.5_GeBr_3_ | ~1.95 | 28 |
|  | [TMAEA]Pb_2_Cl_6_ | ~0.2 | 29 |
|  | (4,4-DFM)_4_AgBiI_8_ | ~1 | 30 |
|  | DMAGeI_3_ | ~12 | this work |


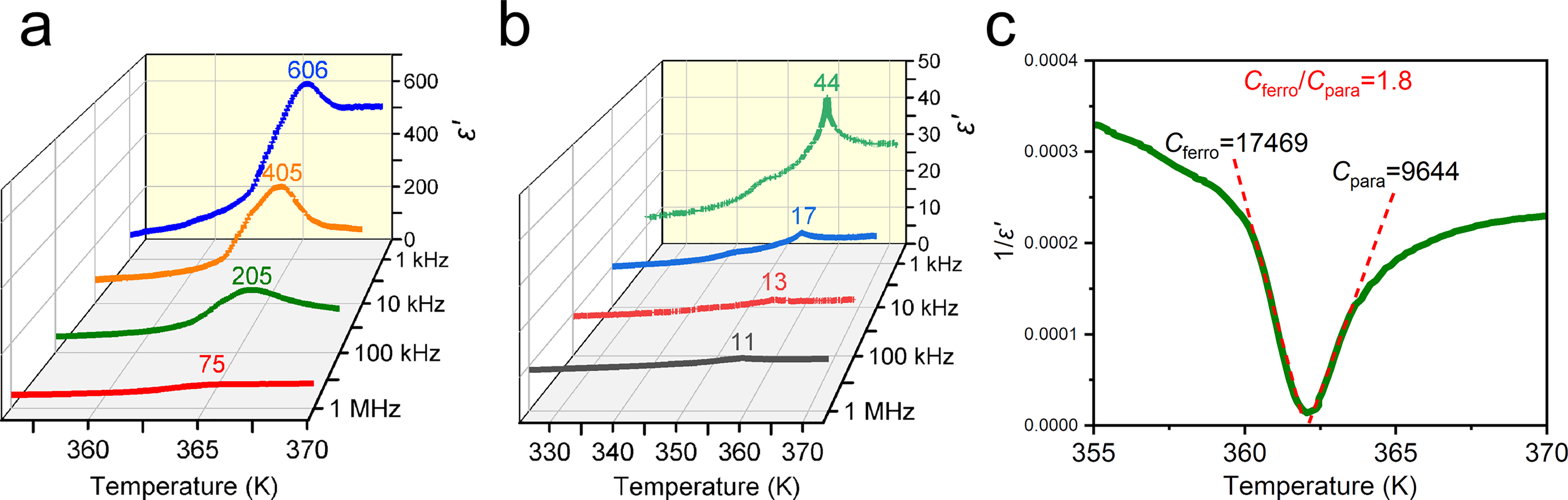


**Supplementary Fig. 11** **| Temperature-dependent dielectric real part (*ε′*) of DMAGeI_3_ measured at various frequencies. a**, along the *a*-axis. **b**, along the *b*-axis. **c**, The fitting to Curie-Weiss law of dielectric anomalies in the vicinity of *T_c_* at 1 kHz.

**
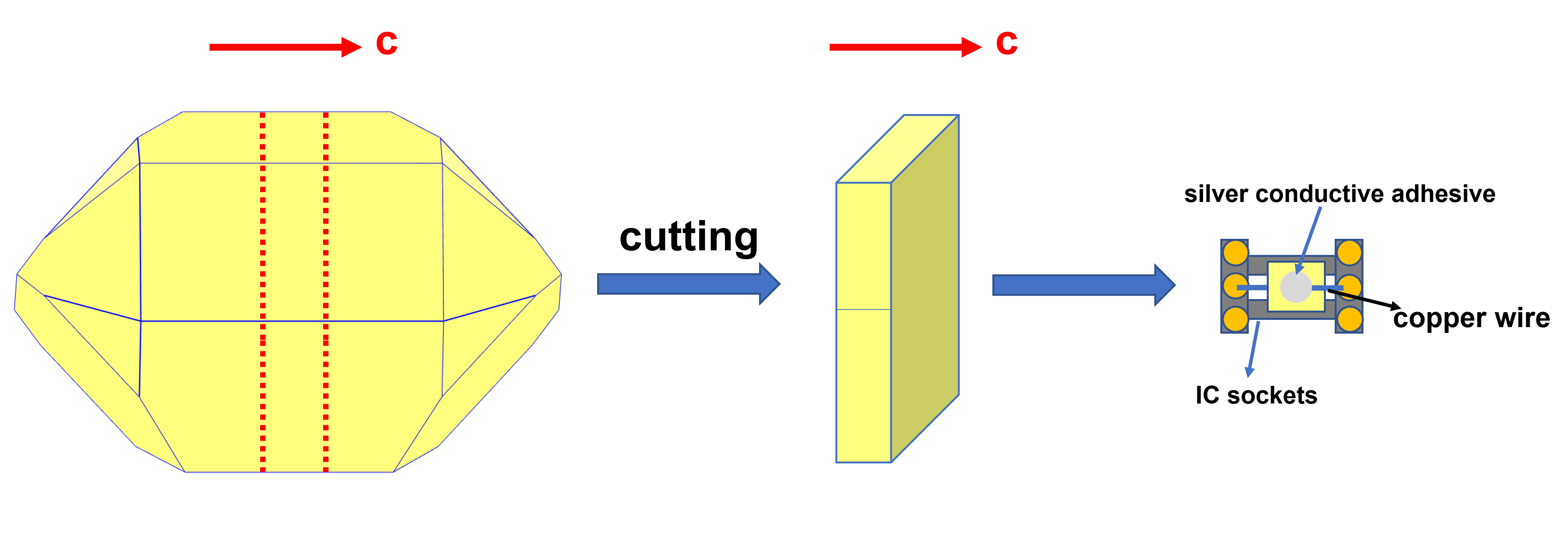
**

**Supplementary Fig. 12 | Crystal process of DMAGeI_3_, cut perpendicular to the *c*-axis.**

**
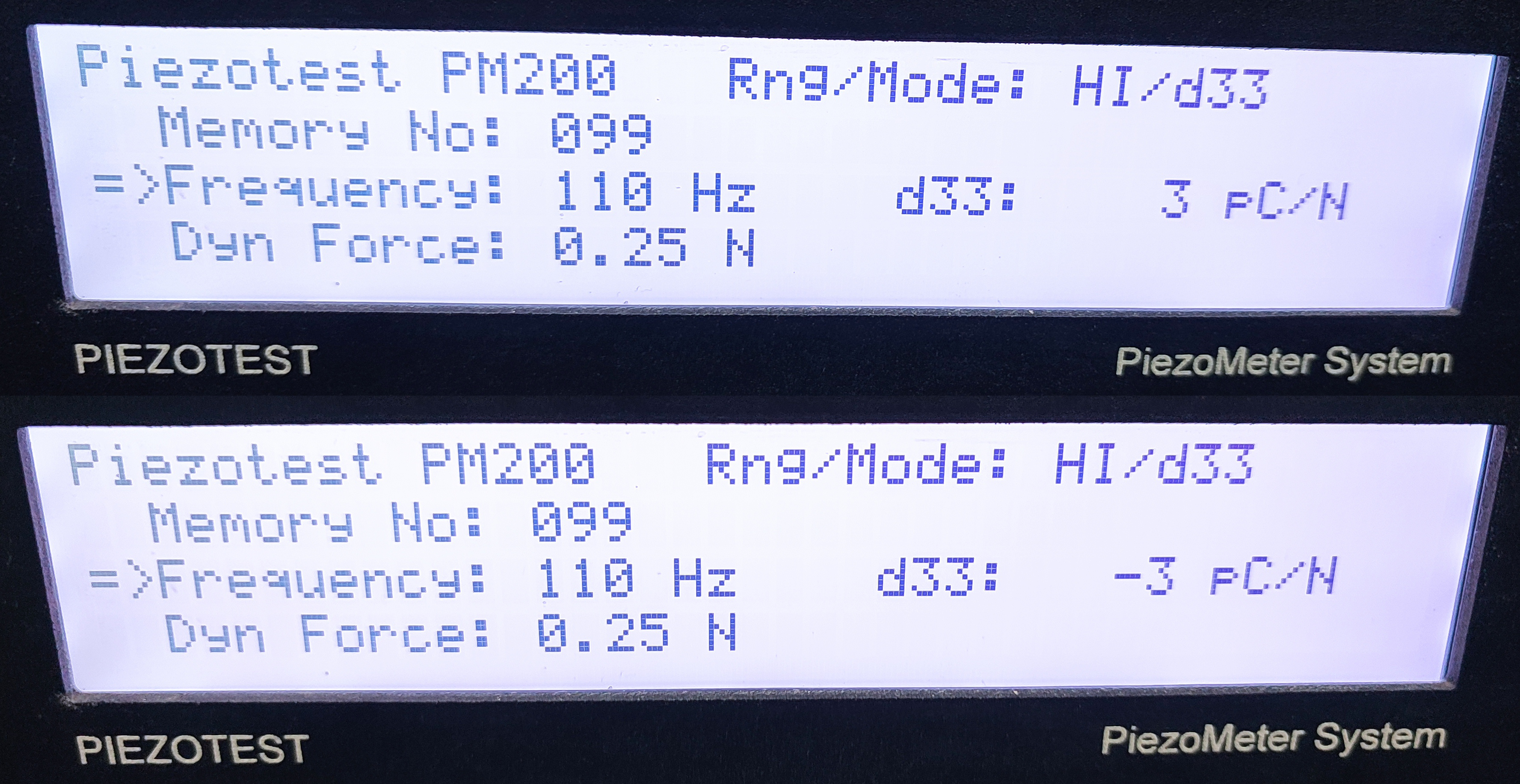
**

**Supplementary Fig. 13 | Photos of *d*_33_ meter.** The maximum *d*_33_ of DMAGeI_3_ is along the vicinity of the [001] direction of the crystal.

**Supplementary Table 5 | Comparison of the maximum dielectric real part (*ε′*) at *T*_c_ of organic-inorganic hybrid perovskite ferroelectrics and DMAGeI_3_ in this work.** BM: benzylammonium; R3HQ: *R*-3-hydroxylquinuclidinium; NPD: *N*-methylpyrrolidinium; 4,4-DCM: 4,4-difluorocyclohexylammonium; RM3HQ: *R*-*N*-methyl-3-hydroxylquinuclidinium; MDABCO: *N*-methyl-*N'*-diazabicyclo[2.2.2]octonium; 4,4-DFM: 4,4-difluoropiperidinium; TMCM: trimethylchloromethyl ammonium.

| Compound | Dielectric | References |
| --- | --- | --- |
| (BM)_2_PbCl_4_ | ~900 (0.5 kHz) | 11 |
| (R3HQ)_4_KCe(NO_3_)_8_ | ~135(1 kHz) | 16 |
| (NPD)_3_Sb_2_Br_9_ | ~460 (1 kHz) | 15 |
| (4,4-DCM)_2_PbI_4_ | ~90 (0.5 kHz) | 32 |
| (RM3HQ)_2_RbLa(NO_3_)_6_ | ~23 (2 kHz) | 33 |
| (C_4_H_9_NH_3_)_2_PbCl_4_ | ~65 (1 kHz) | 34 |
| MDABCO–NH_4_I_3_ | ~15000 (20 Hz) | 35 |
| (3-Pyrrolinium)(CdCl_3_) | ~4600 (0.5 kHz) | 36 |
| (4,4-DFPD)_2_PbI_4_ | ~2200 (0.5 kHz) | 37 |
| [(CH_3_)_2_(F-CH_2_CH_2_)NH]_3_(CdCl_3_)(CdCl_4_) | ~176 (1 kHz) | 38 |
| (S)-3-F-(pyrrolidinium)CdCl_3_ | ~1500 (0.5 kHz) | 39 |
| (4,4-difluoropiperidinium)_4_AgBiI_8_ | ~1700 (0.5 kHz) | 30 |
| TMCM-MnCl_3_ | ~1200 (1 kHz) | 40 |
| BaTiO_3_ | 10000 | 41 |
| KDP | 20000 | 41 |
| TGS | 2000 | 42 |
| Rochelle salt | 4000 | 41 |
| PVDF | 50 | 41 |
| Croconic acid | 850 (30 kHz) | 41 |
| DMAGeI_3_ | 102988(1 kHz) | This work |

**Supplementary Table 6 | Comparison of spontaneous** **polarization *P*_s_ in a variety of materials and DMAGeI_3_ in this work.** TGS: triglycine sulphate; PVDF: Polyvinylidene Fluoride; MDABCO: *N*-methyl-*N'*-diazabicyclo[2.2.2]octonium; QP: Quinuclidinium perrhenate; TMCM: trimethylchloromethyl ammonium; 4,4-DFM: 4,4-difluoropiperidinium;

EATMP: (2-aminoethyl)trimethylphosphanium; dabco: diazabicyclo[2.2.2]octane.

| Compound | *P*_s_ (μC/cm^2^) | Reference |
| --- | --- | --- |
| TGS | 3.8 | 41 |
| BaTiO_3_ | 27 | 42 |
| PVDF | 8 | 42 |
| CsGeI_3_ | 20 | 43 |
| Croconic acid | 20 | 42 |
| MDABCO–NH_4_I_3_ | 22 | 35 |
| TMCM-MnCl_3_ | 4 | 40 |
| TMCM-CdCl_3_ | 6 | 40 |
| QP | 4.5 | 44 |
| Rochelle salt | 0.25 | 42 |
| [NH_4_][Zn(HCOO)_3_] | 0.68 | 45 |
| (3-bromopropylammonium)_2_PbBr_4_ | 4.8 | 46 |
| (4,4-DFPD)_2_PbI_4_ | 10 | 37 |
| [CH_3_(CH_2_)_3_NH_3_]_2_(CH_3_NH_3_)Pb_2_Br_7_ | 3.6 | 21 |
| (C_4_H_9_NH_3_)_2_(C_2_H_5_NH_3_)_2_Pb_3_Br_10_ | 5 | 47 |
| (C_4_H_9_NH_3_)_2_(CH_3_NH_3_)_2_Pb_3_Br_10_ | 2.9 | 48 |
| (EATMP)PbBr_4_ | 0.95 | 49 |
| [Hdabco]BF_4_ | 4.9 | 50 |
| [Hdabco]ClO_4_ | 4 | 51 |
| diisopropylammonium bromide | 23 | 52 |
| [Me_3_NCH_2_CH_2_OH]CdCl_3_ | 17.1 | 53 |
| DMAGeI_3_ | 24.14 | This work |

**Supplementary Table 7 | Comparison of coercive field *E*_c_ in a variety of materials and DMAGeI_3_ in this work**. TGS: triglycine sulphate; PVDF: Polyvinylidene Fluoride; MDABCO: *N*-methyl-*N'*-diazabicyclo[2.2.2]octonium; TMCM: trimethylchloromethyl ammonium; 4,4-DFM: 4,4-difluoropiperidinium; QP: Quinuclidinium perrhenate; EATMP: (2-aminoethyl)trimethylphosphanium; dabco: diazabicyclo[2.2.2]octane.

| Compound | *E*_c_ (kV/cm) | Reference |
| --- | --- | --- |
| TGS | 0.9 | 41 |
| BaTiO_3_ | 10 | 42 |
| PVDF | 500 | 42 |
| CsGeI_3_ | ~40 | 43 |
| Croconic acid | 14 | 42 |
| MDABCO–NH_4_I_3_ | 12 | 35 |
| TMCM-MnCl_3_ | ~27.5 | 40 |
| TMCM-CdCl_3_ | ~9 | 40 |
| QP | ~4 | 44 |
| Rochelle salt | 0.2 | 42 |
| [NH_4_][Zn(HCOO)_3_] | 2.8 | 45 |
| (3-bromopropylammonium)_2_PbBr_4_ | 18.2 | 46 |
| (4,4-DFPD)_2_PbI_4_ | 7.1 | 37 |
| [CH_3_(CH_2_)_3_NH_3_]_2_(CH_3_NH_3_)Pb_2_Br_7_ | 26 | 21 |
| (C_4_H_9_NH_3_)_2_(C_2_H_5_NH_3_)_2_Pb_3_Br_10_ | 8 | 47 |
| (C_4_H_9_NH_3_)_2_(CH_3_NH_3_)_2_Pb_3_Br_10_ | 16 | 48 |
| (EATMP)PbBr_4_ | 7.3 | 49 |
| [Hdabco]BF_4_ | 230 | 50 |
| [Hdabco]ClO_4_ | 83 | 51 |
| DMAGeI_3_ | 0.83-2.2 | This work |


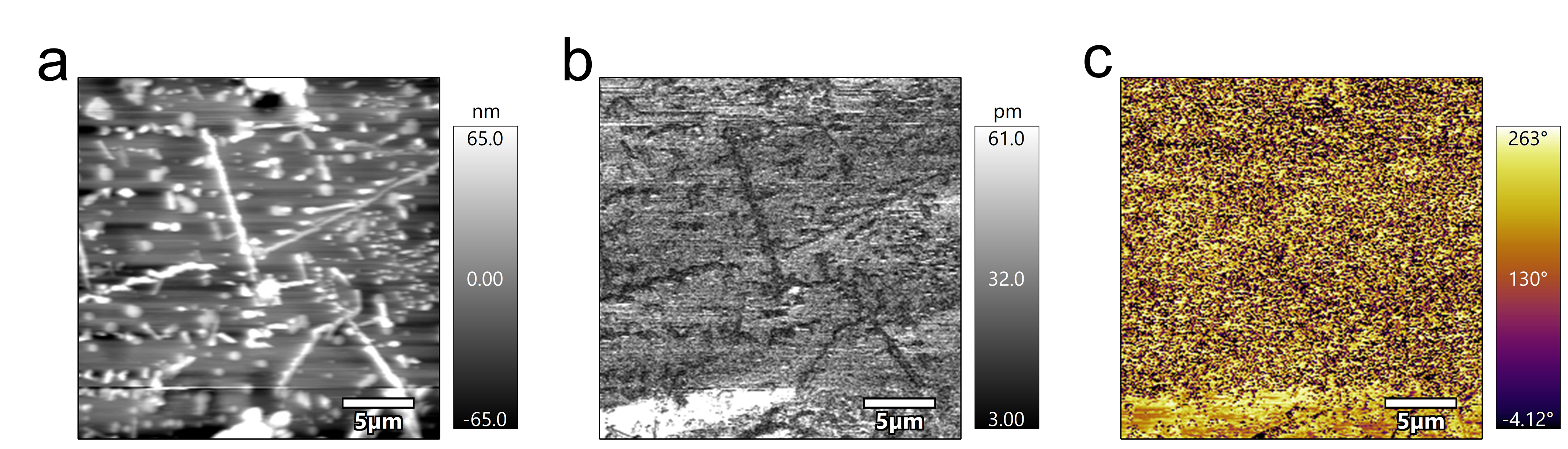


**Supplementary Fig. 14 | Domain structures in the (010) plane of DMAGeI_3_. a**, Topography. **b**, Vertical amplitude images. **c**, The corresponding vertical phase images.


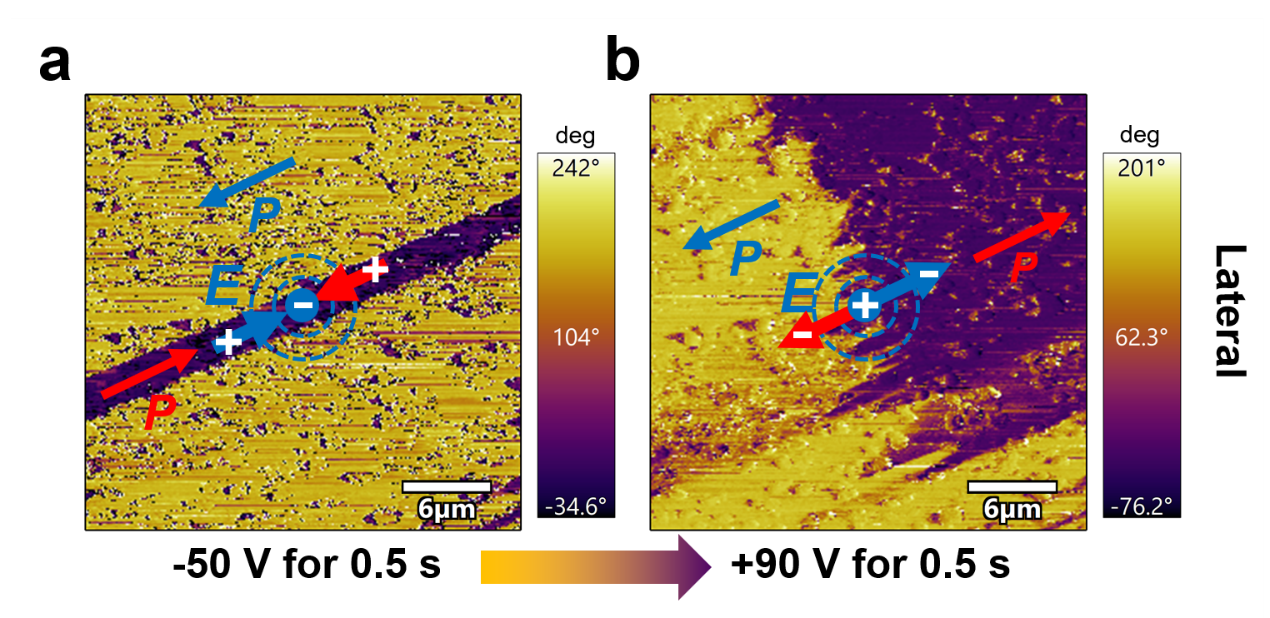


**Supplementary Fig. 15 | The measurements of polarization reversal for DMAGeI_3_ in lateral PFM.** **a**, Phase image the same as Fig. 3i. **b**, Succeeding phase image after applying positive bias of +90 V for 0.5 s.

**
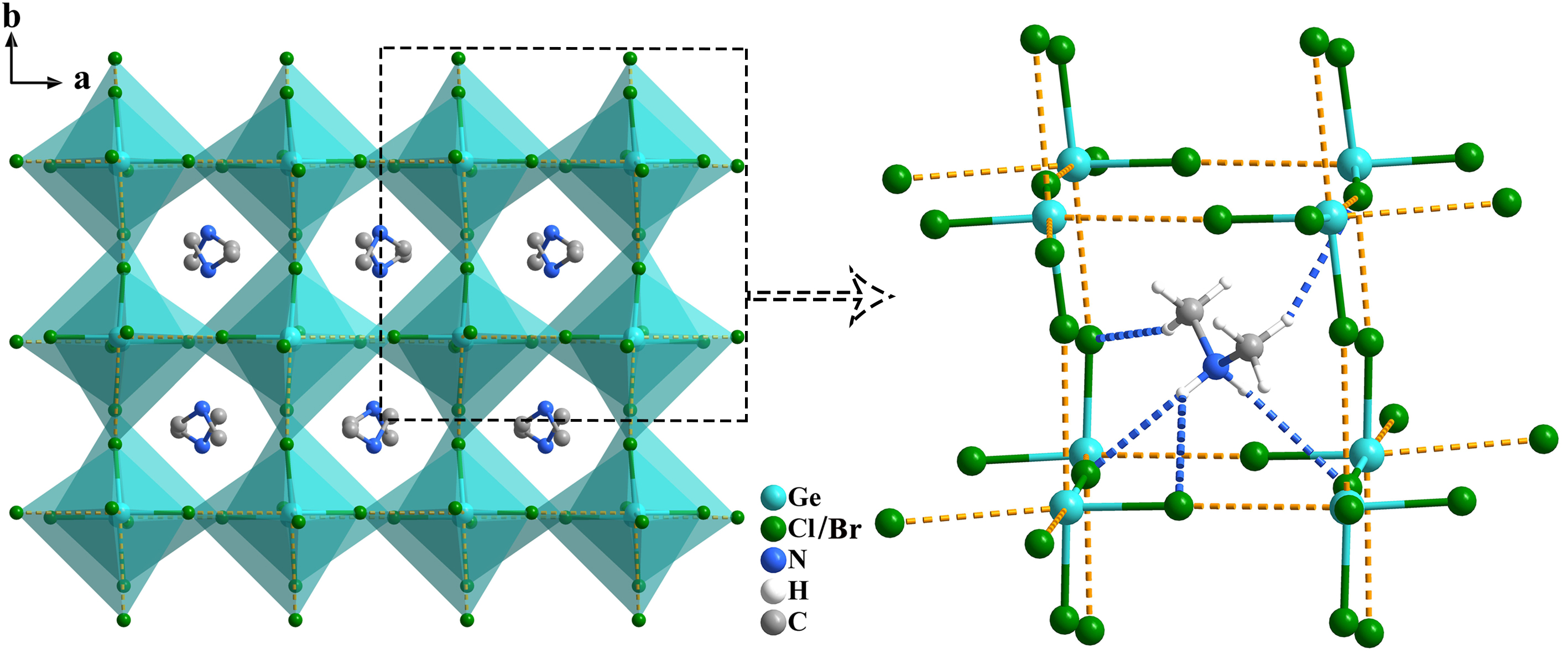
**

**Supplementary Fig. 16 | Structural illustration of the 3D perovskite DMAGeCl_3_ and DMAGeBr_3_ viewed along the *b*-axis.** The isostructural compounds DMAGeCl_3_ and DMAGeBr_3_ crystallize in the orthorhombic system of space group *Pbca* at room temperature. The DMA^+^ are antiparallelly aligned along with the *b*-axis through the hydrogen bonds interaction with the perovskite cage, which causes the disappearance of the molecular polarization. More detailed structural information is summarized in Supplementary Tables 8-9

**Supplementary Table 8 | Crystal data and structure refinement details compound DMAGeCl_3_ and DMAGeBr_3_.**

| Compound | DMAGeCl_3_ | DMAGeBr_3_ |
| --- | --- | --- |
| Temperature/K | 293 | 293 K |
| Empirical formula | C_2_H_8_NGeCl_3_ | C_2_H_8_NGeBr_3_ |
| Formula weight | 225.05 | 358.40 |
| Crystal system | orthorhombic | orthorhombic |
| Space group | *Pbca* | *Pbca* |
| a/Å | 11.5752(8) | 11.9011(8) |
| b/Å | 11.8408(8) | 12.2582(7) |
| c/Å | 12.0003(10) | 12.2854(8) |
| *α*/° | 90 | 90 |
| *β*/° | 90 | 90 |
| *γ*/° | 90 | 90 |
| Volume/Å^3^ | 1644.8(2) | 1792.3(2) |
| Z | 8 | 8 |
| *F* (000) | 880.0 | 1312.0 |
| Radiation | Mo Kα | Mo Kα |
| absorption correction | emiempirical | emiempirical |
| GOF | 1.039 | 1.007 |
| *R*_1_ | 0.0441 | 0.0651 |
| *wR*_2_ | 0.1169 | 0.1713 |

**Supplementary Table 9 | Selected bond lengths (Å) and angles (°) for compounds DMAGeCl_3_ and DMAGeBr_3_ at 293 K, respectively.**

| DMAGeCl_3_ DMAGeBr_3_ | | | | |
| --- | --- | --- | --- | --- |
| Ge01—Cl02 | 2.3249 (11) |  | Ge01—Br02 | 2.4876 (16) |
| Ge01—Cl03 | 2.3150 (12) |  | Ge01—Br03 | 2.4676 (18) |
| Ge01—Cl04 | 2.3454 (12) |  | Ge01—Br04 | 2.5001 (17) |
| N005—C006 | 1.456 (4) |  | N005—C006 | 1.434 (14) |
| N005—C007 | 1.461 (5) |  | N005—C1 | 1.456 (16) |
|  |  |  |  |  |
| Cl02—Ge01—Cl04 | 93.76 (4) |  | Br02—Ge01—Br04 | 94.64 (6) |
| Cl03—Ge01—Cl02 | 94.51 (3) |  | Br03—Ge01—Br02 | 95.47 (6) |
| Cl03—Ge01—Cl04 | 92.16 (5) |  | Br03—Ge01—Br04 | 93.65 (6) |
| C006—N005—C007 | 115.7 (3) |  | C006—N005—C1 | 116.1 (11) |

**Supplementary Note 3 | More details of DFT calculations.**

For comparison, the PBE functional modified for solids (PBEsol) and the D2 Grimme correction for van der Waals interactions have also been tested ^[54, 55]^. The test results of optimized lattice constants indicate that PBE+D3 leads to the best agreement with the experimental data, as compared in Supplementary Fig. 17. Thus, the PBE+D3 is adopted as the default choice in our DFT calculations.

To partition the individual ferroelectric contribution from the GeI_3_ framework and DMA cation, two hypothetic structures are considered. Based on the optimized DMAGeI_3_, the pure GeI_3_ framework is obtained by removing all DMA cations. To keep the charge neutrality, one electron/f.u. is added manually to the GeI_3_ framework. No further structural optimization is performed. Similar process can be done to obtain pure DMA group by removing the GeI_3_ framework and deducting electrons correspondingly. Then their corresponding polarization can be calculated. The direct adding of these two individual contributions is 29.80 μC/cm^2^, very close to the global polarization 29.09 μC/cm^2^. The tiny bias may originate from the interaction between DMA and GeI_3_ parts, which is negligible here.

Furthermore, as presented in Supplementary Fig. 19, the electron density difference, defined as △*ρ = ρ*(DMAGeI_3_)-*ρ*(DMA) where *ρ*(DMAGeI_3_) and *ρ*(DMA) are the electron density of DMAGeI_3_ and DMA with adding hole/f.u., is calculated to support the rationality. It is obvious that the residual electrons only locate at the GeI_3_ framework. Similar situation occurs for △*ρ = ρ*(DMAGeI_3_)-*ρ*(GeI_3_) where *ρ*(GeI_3_) are the electron density of GeI_3_ framework with adding electron/f.u.: the residual electrons only locate at the DMA group. Therefore, our treatment of individual parts can restore the charge distribution in original DMAGeI_3_.

**Supplementary Fig. 17 | The test of functionals and van der Waals corrections.** △*l*=*l*-*l*_exp_, where *l* and *l*_Exp_ are the lattice constants obtained from DFT calculation and experimental data.

**Supplementary Fig. 18 | DFT electronics properties of DMAGeI_3_.** Left: band structure. Inset: the schematic of Brillouin zone and *k*-path for the DMAGeI_3_. The direct band gap at Γ point is 2.79 eV. Right: atom-projected DOS. The bands near Fermi level are mainly contributed by the GeI_3_ framework.


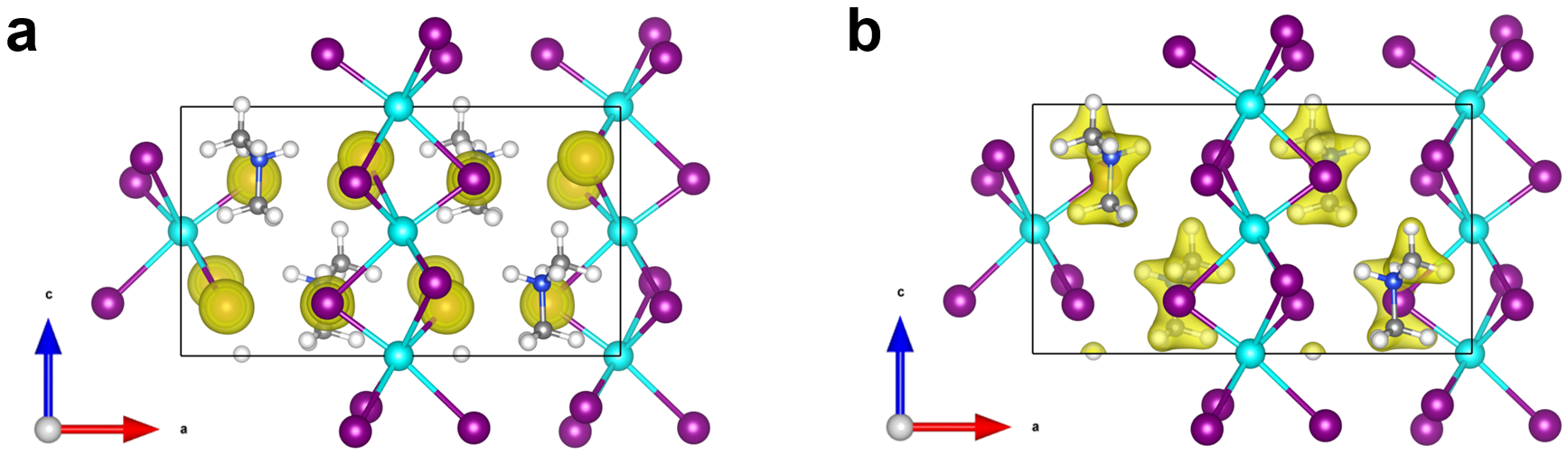


**Supplementary Fig. 19 | Electron density difference between DMAGeI_3_ and extracted parts with holes/electrons added. a,** Between the DMAGeI_3_ and the pure DMA group. **b,** Between the DMAGeI_3_ and the GeI_3_ framework. The isosurfaces are set to 0.1 *e*/Å^3^.

**Supplementary Note 4 | More details of SHG calculations.**

The SHG intensity can be estimated via $I\propto(P^{2\omega})^{2}=P_{a}^{2}+P_{b}^{2}+P_{c}^{2}$, where *P*^2^*^^* is the second harmonic polarization generated by the electric field of incident light with angular frequency. *a*/*b*/*c* are perpendicular crystalline axes here. For the LTP DMAGeI_3_ with *Pna*2_1_ space group, $P^{2\omega}$ can be given by ^[56]^:

$\left(\begin{matrix} P_{a} \\ P_{b} \\ P_{c} \end{matrix}\right)=2\times\left(\begin{matrix} 0 & 0 & 0 & 0 & d_{15} & 0 \\ 0 & 0 & 0 & d_{24} & 0 & 0 \\ d_{31} & d_{32} & d_{33} & 0 & 0 & 0 \end{matrix}\right)\left(\begin{matrix} E_{a}^{2} \\ E_{b}^{2} \\ E_{c}^{2} \\ 2E_{b}E_{c} \\ \begin{matrix} 2E_{a}E_{c} \\ 2E_{a}E_{b} \end{matrix} \end{matrix}\right)=2\times\left(\begin{matrix} 2d_{15}E_{a}E_{c} \\ 2d_{24}E_{b}E_{c} \\ d_{31}E_{a}^{2}+d_{32}E_{b}^{2}+d_{33}E_{c}^{2} \end{matrix}\right)$, (1)

where *d_ij_*’s are the elements of second-harmonic nonlinear optical susceptibility tensor, and *E_i_* is component of electric field of incident light.

As presented in Supplementary Fig. 20a, we consider the *bc* plane which is the most possible one in our experimental setup. Then the propagation direction of incident light is parallel to the *a* axis. The electric field of incident light can be written as *E*=(*E_a_*, *E_b_*, *E_c_*)=*E*(0, cos*ϕ*, sin*ϕ*), where *j* is the angle between the *b*-axis and electric field. Then, the SHG intensity of DMAGeI_3_ is:

. (2)

For KDP with $I\overline{4}2d$ space group, $P^{2\omega}$ can be given by ^[56]^:

$\left(\begin{matrix} P_{a} \\ P_{b} \\ P_{c} \end{matrix}\right)=2\times\left(\begin{matrix} 0 & 0 & 0 & d_{14} & 0 & 0 \\ 0 & 0 & 0 & 0 & d_{14} & 0 \\ 0 & 0 & 0 & 0 & 0 & d_{36} \end{matrix}\right)\left(\begin{matrix} E_{a}^{2} \\ E_{b}^{2} \\ E_{c}^{2} \\ 2E_{b}E_{c} \\ \begin{matrix} 2E_{a}E_{c} \\ 2E_{a}E_{b} \end{matrix} \end{matrix}\right)=2\times\left(\begin{matrix} 2d_{14}E_{b}E_{c} \\ 2d_{14}E_{a}E_{c} \\ 2d_{36}E_{a}E_{b} \end{matrix}\right)$. (3)

Then, the SHG intensity of KDP on its *bc* plane can be expressed as:

. (4)

By using the *exciting* package ^[57]^, the nonlinear optical susceptibility tensor *d* can be calculated. The calculated values of *d_ij_*’s for DMAGeI_3_ and KDP are compared in Supplementary Fig. 20a. Note that our calculated *d*_36_=0.40 pm/V of KDP is close to its experimental value 0.38 pm/V ^[58]^, implying the reliability of calculation. The derived SHG anisotropy of the DMAGeI_3_ also agrees well with the experimental measurement as presented in Supplementary Fig. 20b.

**Supplementary Fig. 20 | SHG analysis. a**, The calculated values of main *d_ij_*’s for DMAGeI_3_ and KDP. **b**, The normalized SHG intensity anisotropy obtained from the experimental measurement (Exp.) and our calculation (Theory) for DMAGeI_3_.

**Supplementary References**

[1] García-Fernández, A. *et al*. Phase transition, dielectric properties, and ionic transport in the [(CH_3_)_2_NH_2_]PbI_3_ organic-inorganic hybrid with 2H-hexagonal perovskite structure. *Inorg. Chem.* **56**, 4918-4927 (2017).

[2] Ju., D. *et al*. Reversible band gap narrowing of Sn-based hybrid perovskite single crystal with excellent phase stability. *Angew. Chem. Int. Ed.* **57,** 14868-14872(2018).

[3] Han, X.-B. *et al*. Structural descriptors to correlate Pb ion displacement and broadband emission in 2D halide perovskites. *J. Am. Chem. Soc.* **144**, 18595-18606 (2022).

[4] Dunn, M. H., Ebrahimzadeh, M. Parametric generation of tunable light from continuous-wave to femtosecond pulses. *Science*, **286**, 1513-1517 (1999).

[5] Ezhov, D. M. *et al*. In SHG in gamma-Ga_2_S_3_ powder, 13th international conference on atomic and molecular pulsed lasers (AMPL), Tomsk, RUSSIA, Sep 10-15 (2018); Tomsk, RUSSIA, 2017.

[6] Eimerl, D. *et al*. Optical, mechanical, and thermal-properties of barium borate. *J. Appl. Phys.* **62**, 1968-1983 (1987).

[7] Chen, C. T. *et al*. New nonlinear-optical crystal-LiB_3_O_5_. *J. Opt. Soc. Am. B*. **6**, 616-621 (1989).

[8] Borsutzky, A., Brunger, R., Huang, C. & Wallenstein, R. Harmonic and sum-frequency generation of pulsed laser-radiation in BBO, LBO and KDP. *Appl. Phys. B: Photophys. Laser Chem.* **52**, 55-62 (1991).

[9] Huang, C.-R. *et al*. A multiaxial lead-free two-dimensional organic-inorganic perovskite ferroelectric. *Natl. Sci. Rev.* **8**, nwaa232 (2020).

[10] Shi, P.-P. *et al*. Two-dimensional organic–inorganic perovskite ferroelectric semiconductors with fluorinated aromatic spacers. *J. Am. Chem. Soc.* **141**, 18334-18340 (2019).

[11] Fu, D. *et al*. Multiaxial ferroelectricity and ferroelasticity in a chiral perovskite. *Chem. Mater.* **34**, 3518-3524 (2022).

[12] Ai, Y. *et al*. Unprecedented ferroelectricity and ferromagnetism in a Cr^2+^-based two-dimensional hybrid perovskite. *Angew. Chem., Int. Ed.* **61**, e202206034 (2022).

[13] Hu, Z.-B. *et al*. An effective strategy of introducing chirality to achieve multifunctionality in rare-earth double perovskite ferroelectrics. *Small Methods*. **6**, 2200421 (2022).

[14] Yang, C.-K. *et al*. The first 2D homochiral lead iodide perovskite ferroelectrics: [R- and S-1-(4-Chlorophenyl)ethylammonium]_2_PbI_4_. *Adv. Mater.* **31**, 1808088 (2019).

[15] Sun, Z. *et al*. Exploring a lead-free semiconducting hybrid ferroelectric with a zero-dimensional perovskite-like structure. *Angew. Chem., Int. Ed.* **55**, 11854-11858 (2016).

[16] Shi, C. *et al*. Two-dimensional organic-inorganic hybrid rare-earth double perovskite ferroelectrics. *J. Am. Chem. Soc.* **142**, 545-551 (2020).

[17] Li, L. *et al*. A potential Sn-based hybrid perovskite ferroelectric semiconductor. *J. Am. Chem. Soc.* **142**, 1159-1163 (2020).

[18] Liu, X. *et al*. Giant room temperature electrocaloric effect in a layered hybrid perovskite ferroelectric: [[(CH](https://www.x-mol.com/paperRedirect/1441527752008986624)[3](https://www.x-mol.com/paperRedirect/1441527752008986624)[)](https://www.x-mol.com/paperRedirect/1441527752008986624)[2](https://www.x-mol.com/paperRedirect/1441527752008986624)[CHCH](https://www.x-mol.com/paperRedirect/1441527752008986624)[2](https://www.x-mol.com/paperRedirect/1441527752008986624)[NH](https://www.x-mol.com/paperRedirect/1441527752008986624)[3](https://www.x-mol.com/paperRedirect/1441527752008986624)[]](https://www.x-mol.com/paperRedirect/1441527752008986624)[2](https://www.x-mol.com/paperRedirect/1441527752008986624)[PbCl](https://www.x-mol.com/paperRedirect/1441527752008986624)[4](https://www.x-mol.com/paperRedirect/1441527752008986624). *Nat. Commun.* **12**, 5502 (2021).

[19] Tang, Y.-Y. *et al*. Three-dimensional lead bromide hybrid ferroelectric realized by lattice expansion. *J. Am. Chem. Soc.* **142**, 19698-19704 (2020).

[20] Hua, X.-N. *et al*. A room-temperature hybrid lead iodide perovskite ferroelectric. *J. Am. Chem. Soc.* **140**, 12296-12302 (2018).

[21] Li, L. *et al*. Two-dimensional hybrid perovskite-type ferroelectric for highly polarization-sensitive shortwave photodetection. *J. Am. Chem. Soc.* **141**, 2623-2629 (2019).

[22] Yao, Y. *et al*. High-curie temperature multilayered hybrid double perovskite photoferroelectrics induced by aromatic cation alloying. *J. Am. Chem. Soc.* **143**, 15900-15906 (2021).

[23] Deng, B.-B. *et al*. The first salicylaldehyde schiff base organic-inorganic hybrid lead iodide perovskite ferroelectric. *Chem. Commun.* **58**, 2192-2195 (2022).

[24] Maczka, M. *et al*. Three-dimensional perovskite methylhydrazinium lead chloride with two polar phases and unusual second-harmonic generation bistability above room temperature. *Chem. Mater.* **32**, 4072-4082 (2020).

[25] Maczka, M. *et al*. Methylhydrazinium lead bromide: Noncentrosymmetric three-dimensional perovskite with exceptionally large framework distortion and green photoluminescence. *Chem. Mater.* **32**, 1667-1673 (2020).

[26] Dang, Y. *et al*. Crystallographic-investigations into properties of acentric hybrid perovskite single crystals NH(CH_3_)_3_SnX_3_ (X = Cl, Br). *Chem. Mater.* **28**, 6968-6974(2016).

[27] Stoumpos, C. C. *et al*. Hybrid germanium iodide perovskite semiconductors: Active lone pairs, structural distortions, direct and indirect energy gaps, and strong nonlinear optical Properties. *J. Am. Chem. Soc.* **137**, 6804-6819 (2015).

[28] Liu, Y. *et al*. Hybrid germanium bromide perovskites with tunable second harmonic generation. *Angew. Chem., Int. Ed.* **61**, e202208875 (2022).

[29] Zhang, H.-Y *et al*. A three-dimensional lead halide perovskite-related ferroelectric. *J. Am. Chem. Soc.* **142,** 4604-4608 (2020).

[30] Wang, C.-F. *et al*. Centimeter-sized single crystals of two-dimensional hybrid iodide double perovskite (4,4-Difluoropiperidinium)_4_AgBiI_8_ for high-temperature ferroelectricity and efficient X-ray detection. *Adv. Funct. Mater.* **31**, 2009457 (2021).

[31] Liao, W.-Q. *et al*. A lead-halide perovskite molecular ferroelectric semiconductor. *Nat. Commun.* **6**, 7338 (2015).

[32] Sha, T.-T. *et al*. Fluorinated 2D lead iodide perovskite ferroelectrics. *Adv. Mater.* **31**, 1901843 (2019).

[33] Shi, C. *et al*. Large piezoelectric response in hybrid rare-earth double perovskite relaxor ferroelectrics. *J. Am. Chem. Soc.* **142,** 9634-9641 (2020).

[34] Ji, C., Wang, S., Li, L., Sun, Z., Hong, M., Luo, J. The first 2D hybrid perovskite ferroelectric showing broadband white-light emission with high color rendering index. *Adv. Funct. Mater.* **29**, 1805038 (2019).

[35] Ye, H.-Y. *et al*. Metal-free three-dimensional perovskite ferroelectrics. *Science.* **361**, 151-155 (2018).

[36] Ye, H.-Y., Zhang, Y., Fu, D.-W. & Xiong, R.-G. An above-room-temperature ferroelectric organo-metal halide perovskite: (3-Pyrrolinium)(CdCl_3_). *Angew. Chem., Int. Ed.* **53**, 11242-11247 (2014).

[37] Zhang, H.-Y. *et al*. Observation of vortex domains in a two-dimensional lead iodide perovskite ferroelectric. *J. Am. Chem. Soc.* **142**, 4925-4931 (2020).

[38] Wang, Z.-X. *et al*. Fluoridation achieved antiperovskite molecular ferroelectric in (CH_3_)_2_(F-CH_2_CH_2_)NH_3_(CdCl_3_)(CdCl_4_). *J. Am. Chem. Soc.* **141**, 4372-4378 (2019).

[39] Tang, Y.-Y. *et al*. H/F-substitution-induced homochirality for designing high-*T*c molecular perovskite ferroelectrics. *Adv. Mater.* **31**, 1902163 (2019).

[40] You, Y.-M. *et al*. An organic-inorganic perovskite ferroelectric with large piezoelectric response. *Science.* **357,** 306-309 (2017).

[41] Hoshino, S., Mitsui, T., Jona, F., Pepinsky, R. Dielectic and thermal study of tri-glycine sulfate and tri-glycine fluoberyllate. *Phys. Rev.* **107,** 1255-1258 (1957).

[42] Horiuchi, S.; Tokura, Y. Organic ferroelectrics. *Nat. Mater.* **7**, 357-366 (2008).

[43] Zhang, Y. *et al*. Ferroelectricity in a semiconducting all-inorganic halide perovskite. *Sci. Adv.* **8**, eabj5881 (2022).

[44] Harada, J. *et al*. Directionally tunable and mechanically deformable ferroelectric crystals from rotating polar globular ionic molecules. *Nat. Chem.* **8**, 946-952 (2016).

[45] Xu, G.-C. *et al*. Disorder-order ferroelectric transition in the metal formate framework of NH_4_Zn(HCOO)_3_. *J. Am. Chem. Soc.* **132**, 9588-9590 (2010).

[46] Ji, C. *et al*. Ferroelectricity-driven self-powered ultraviolet photodetection with strong polarization sensitivity in a two-dimensional halide hybrid perovskite. *Angew. Chem., Int. Ed.* **59**, 18933-18937 (2020).

[47] Ji, C. *et al*. 2D Hybrid perovskite ferroelectric enables highly sensitive X-ray detection with low driving voltage. *Adv. Funct. Mater.* **30**, 1905529 ( 2020).

[48] Li, L. *et al*. Tailored engineering of an unusual (C_4_H_9_NH_3_)_2_(CH_3_NH_3_)_2_Pb_3_Br_10_ two-dimensional multilayered perovskite ferroelectric for a high-performance photodetector. *Angew. Chem., Int. Ed.* **56**, 12150-12154 (2017).

[49] Zhang, H.-Y. *et al*. Large electrostrictive coefficient in a two-dimensional hybrid perovskite ferroelectric. *J. Am. Chem. Soc.* **143**, 1664-1672 (2021).

[50] Shi, P.-P. *et al*. De novo discovery of Hdabco BF_4_ molecular ferroelectric thin film for nonvolatile low-voltage memories. *J. Am. Chem. Soc.* **139,** 1319-1324 (2017).

[51] Tang, Y.-Y. *et al*. Ultrafast polarization switching in a biaxial molecular ferroelectric thin film: Hdabco ClO_4_. *J. Am. Chem. Soc.* **138**, 15784-15789 (2016).

[52] Fu, D. W. *et al.* Diisopropylammonium bromide is a high-temperature molecular ferroelectric crystal. *Science*, **339**, 425-428 (2013).

[53] Swati D. *et al.* Neutral 1D perovskite-type ABX_3_ ferroelectrics with high mechanical energy harvesting performance. *Chem. Mater.* **32**, 8333−8341(2020).

[54] John P P., *et al*. [Restoring the density-gradient expansion for exchange in solids and surfaces](http://arxiv.org/abs/0711.0156). *Phys. Rev. Lett.* **100**, 136406 (2008).

[55] [Stefan G](https://onlinelibrary.wiley.com/action/doSearch?ContribAuthorRaw=Grimme%2C+Stefan). Semiempirical GGA-type density functional constructed with a long-range dispersion correction. *J. Comput. Chem.* **27**, 1787 (2006).

[56] Sutherland R. L. Handbook of nonlinear optics. CRC Press (2003). And nonlinear susceptibility tensor can also be obtained via <https://www.cryst.ehu.es/cgi-bin/cryst/programs/tensor.pl>;

[57] A. Gulans, S. *et al*. exciting: a full-potential all-electron package implementing density-functional theory and many-body perturbation theory. *J. Phys.: Condens. Matter.* **26**, 363202 (2014).

[58] R.C. Eckardt. *et al*. Byer: Absolute and relative nonlinear optical coefficients of KDP, KD*P, BaB_2_O_4_, LiIO_3_, MgO: LiNbO_3_, and KTP measured by phase-matched second-harmonic generation. *IEEE J. Quant. Electr.* **26**, 922-933 (1990).
